# Role of Antioxidant Systems and Heat Shock Response in Aquatic Animals Under Multistress Conditions

**DOI:** 10.3390/antiox15080917

**Published:** 2026-07-23

**Authors:** Konstantinos Feidantsis, Marina Minari, Víctor Cubillos, Peter D. Dijkstra, Olivia D. K. Buzinski, Daniel C. Moreira, Marcelo Hermes-Lima

**Affiliations:** 1 Department of Fisheries and Aquaculture, School of Agricultural Sciences, University of Patras, GR-26504 Mesolonghi, Greece; 2 Departamento de Biologia Celular, Universidade de Brasília, Brasília 70910-900, Brazil; marina.carvalho@aluno.unb.br; 3 Instituto de Ciencias Marinas y Limnológicas, Universidad Austral de Chile, Valdivia 5090000, Chile; victor.cubillos@uach.cl; 4 Department of Biology, Central Michigan University, Mount Pleasant, MI 48859, USA; dijks1p@cmich.edu (P.D.D.); buzin1od@cmich.edu (O.D.K.B.); 5 Faculdade de Medicina, Universidade de Brasília, Brasília 70910-900, Brazil; moreiradc@unb.br

**Keywords:** Hsp, antioxidants, marine environment, stress response, redox metabolism

## Abstract

The multistress concept is a recent approach developed to better understand the effects of environmental stress in animals under real-world conditions. In nature, animals are exposed to simultaneous environmental fluctuations on daily and seasonal time scales, and interactions among stressors can produce antagonistic, additive, or synergistic physiological responses. Consequently, studies examining only one stressor under controlled laboratory conditions may fail to reflect responses in natural habitats. This review discusses the effects of multiple stressors on redox metabolism and heat shock protein (HSP) responses, focusing on aquatic environments. We discuss the multistress approach in laboratory and field studies, highlighting major abiotic stressors such as salinity changes, solar radiation, temperature, and low oxygen availability, including hypoxia and aerial exposure. The effects of multiple stressors on HSPs and antioxidants are summarized using examples from the literature. Finally, the role of social stress and life history stage in shaping responses to multiple abiotic stressors is considered. Overall, the review highlights the application of the multistress approach in laboratory and field experiments, emphasizing key stress responses involving endogenous antioxidants and HSPs, both initiated by reactive oxygen species (ROS).

## 1. General Introduction

This review article examines how animals respond to various environmental stressors—such as temperature fluctuations, hypoxia, and UV radiation—by focusing on free radical metabolism (hereafter referred to as redox metabolism) and the heat shock response. Until two decades ago, the vast majority of studies on oxidative stress focused on analyzing a single abiotic stressor, occasionally examining the timeline of physiological events. Such an approach is often excessively artificial, as stress intensity typically fluctuates over time and multiple abiotic and biotic stressors may interact simultaneously, as discussed in detail throughout this article.

To illustrate this, let us examine a study conducted by our group two decades ago [1]. This research investigated the effects of anoxia exposure on oxidative stress and antioxidant variables in goldfish organs. We observed that eight hours of anoxia induced the activity of two antioxidant enzymes—one in the brain and another in the liver. Furthermore, lipid peroxidation increased between 1 h and 14 h during post-anoxic reoxygenation in both tissues. While this was one of the first studies to detect reoxygenation damage in fish and highlight the role of endogenous antioxidants in mitigating such damage, it possessed a significant design limitation: it was too artificial.

In nature, fish do not transition “instantly” from a normoxic environment to one of near-total anoxia; instead, this depletion occurs gradually in the wild. Similarly, reoxygenation is typically a progressive process. For instance, while ice-covered waters in winter—the typical environment for feral goldfish in Eurasia [2,3]—tend to have significantly less oxygen, this depletion occurs over days or weeks rather than minutes. Consequently, oxygen returns slowly as the ice melts.

Moreover, the goldfish experiment was conducted at a fixed temperature of 20 °C [1]. In nature, temperature rarely remains stable for 22 h (the duration of the 8 h anoxia plus 14 h reoxygenation period), and fish enduring hypoxia in winter are subjected to much colder temperatures. Such thermal variations likely influence the impact of anoxia/reoxygenation on goldfish redox metabolism in ways that remained uninvestigated at the time. Other factors, such as water pH or pollutants, could also influence oxidative stress responses.

Over the past 15 years, researchers studying various vertebrate and invertebrate species have increasingly addressed these complexities, moving away from the traditional focus on single stressors toward an experimental setup based on a combination of stresses (example: temperature changes and hypoxia). Such strategy is the so-called multistress approach, which is introduced in detail in Section 2. In this review we examined how the redox metabolism and heat shock response (HSR) in animals is affected by a combination of stresses. We analyzed studies conducted both in laboratory settings (incorporating more than one stressor) and in situ (employing animal samples directly in nature), where environmental conditions are inherently multifactorial. This review is structured to cover the effects of stressors on HSPs and redox metabolism across a wide range of taxa, primarily focusing on abiotic stresses affecting aquatic animals (freshwater, estuarine and marine), although a few examples of terrestrial species are included. Finally, we discuss the impact of social stress—a form of biotic stress—on the redox metabolism of aquatic animals.

## 2. The Multistress Concept in Ecophysiology

Studies on animal responses to multiple stressors have been common for over a century in fields such as toxicology and ecology [4,5,6], but this is a relatively new concept in ecophysiology and comparative physiology [7,8,9]. As a result, different terms are often used across disciplines to describe the same concepts [6]. For conceptual clarity, we compiled a Glossary of key terms in multistress research with definitions on how they are used throughout this review.

**Glossary:** Definitions of cross-protection, cross-tolerance, and cross-talk were based on Rodgers et al. [8,9]; experimental approaches were defined according to Diamond [10]; physiological effects were adapted from Todgham and Stillman [11] and Gunderson et al. [12]; and the definitions of multistress and stressor were proposed by the authors based on terminology commonly used in the literature and adapted to the context of this review.

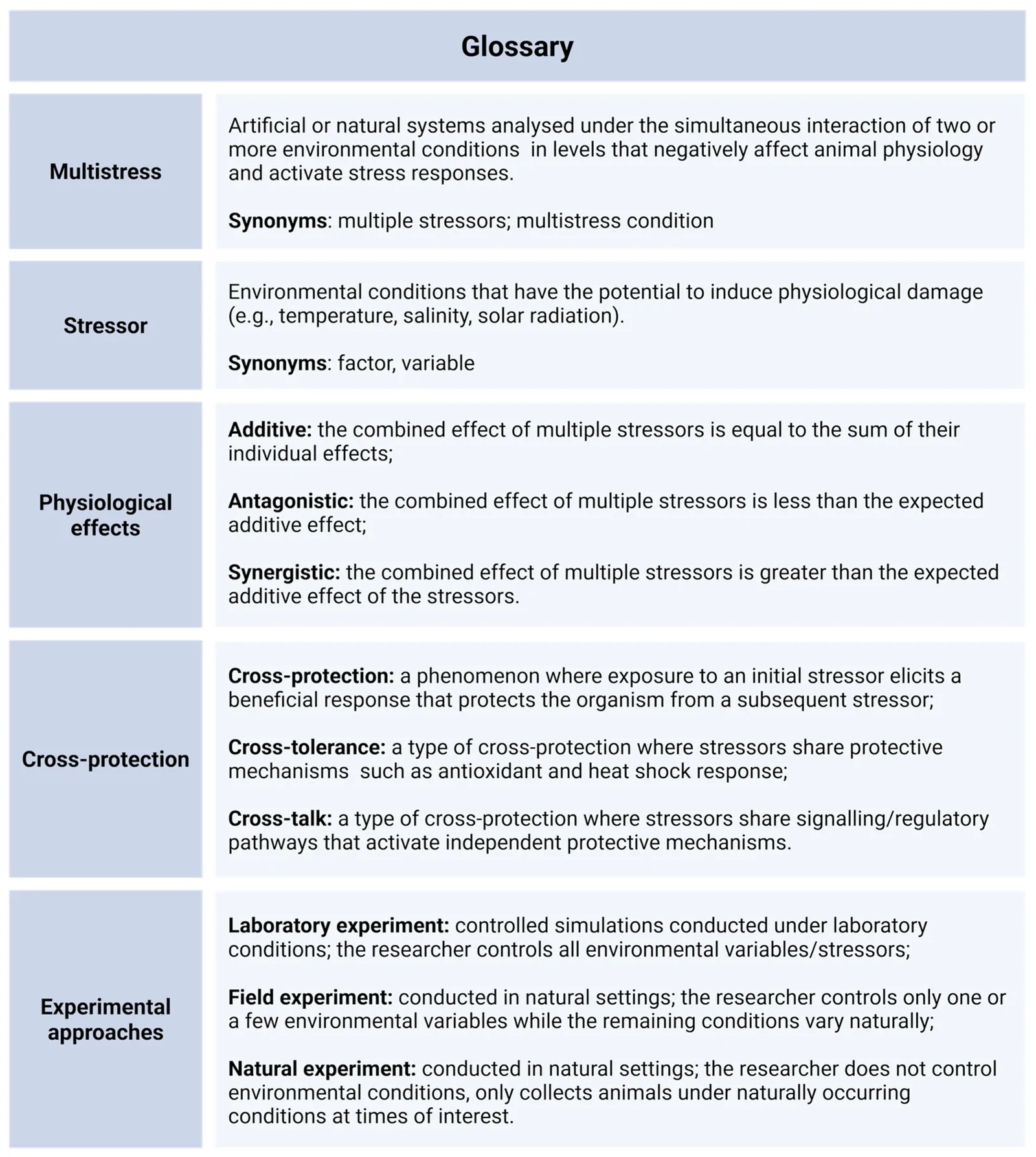



In ecophysiology, researchers have traditionally subjected organisms to constant conditions within a given treatment. However, aquatic environments are highly variable through space, time, and scales, exposing their fauna to a plethora of abiotic fluctuations. Some of the environmental conditions that can act as stressors (see Glossary) in aquatic systems are:

(i) Temperature fluctuations [13];
(ii) Variations in oxygen availability—such as hypoxic conditions in dead zones and tide-influenced coastal regions [14];
(iii) High UV-radiation incidence in shallow waters [15,16,17,18];
(iv) Salinity shifts, particularly in estuarine environments [19,20];
(v) Changes in pH levels, often driven by ocean acidification [21,22].

These abiotic factors can fluctuate on daily and/or seasonal scales [22,23,24] and be highly influenced by seasonality and anthropogenic factors. Additionally, aquatic organisms are exposed to other significant stressors, including:

(i) Pollutants such as heavy metals, microplastics, and organic contaminants [25,26];
(ii) Competition and predation pressures [27];
(iii) The presence of invasive species disrupting local ecosystems [28,29];
(iv) Physical disturbances caused by waves, winds, and rain [30,31].

Organismal responses to such environmental challenges and their impact on performance have been studied through countless experimental approaches in laboratory conditions, typically considering just one abiotic factor (e.g., temperature) as a predictive variable while keeping all others constant [32,33,34]. However, even laboratory studies designed to closely approximate these natural dynamics—by incorporating multiple stressors into the analysis—often fail to capture realistic exposures observed in nature. For example, mean environmental conditions—often used in laboratory settings—ignore peaks that may have enormous detrimental consequences, while exposure to constant extreme stress may facilitate adaptation to these conditions [12,35,36]. Thus, the use of biological models in static experiments only hinders the accuracy of predicting the impact of organisms under natural conditions, especially under the continuously changing climate/weather conditions [37,38]. Currently, there is a need to determine the realistic effect of multiple stressors on the cellular and physiological response of different organisms in natural settings, where multiple environmental conditions fluctuate simultaneously [37]. Field studies complemented with controlled laboratory exposures that simulate field conditions can be a useful tool to reveal sophisticated physiological responses of animals to multiple interacting stressors [39,40,41,42,43,44,45].

### 2.1. Characterizing Stressor Interactions

Research regarding multistress effects is of high importance in the field of environmental sciences, such as ecophysiology. Typically, such studies expose organisms to elevated levels of each stressor to maximize and observe effects in short-term settings, while simultaneously subjecting a separate cohort to the combined action of all examined factors [12]. This implies that the effect of more than one stressor acting simultaneously may be completely different from those observed independently [46].

**Figure 1:**
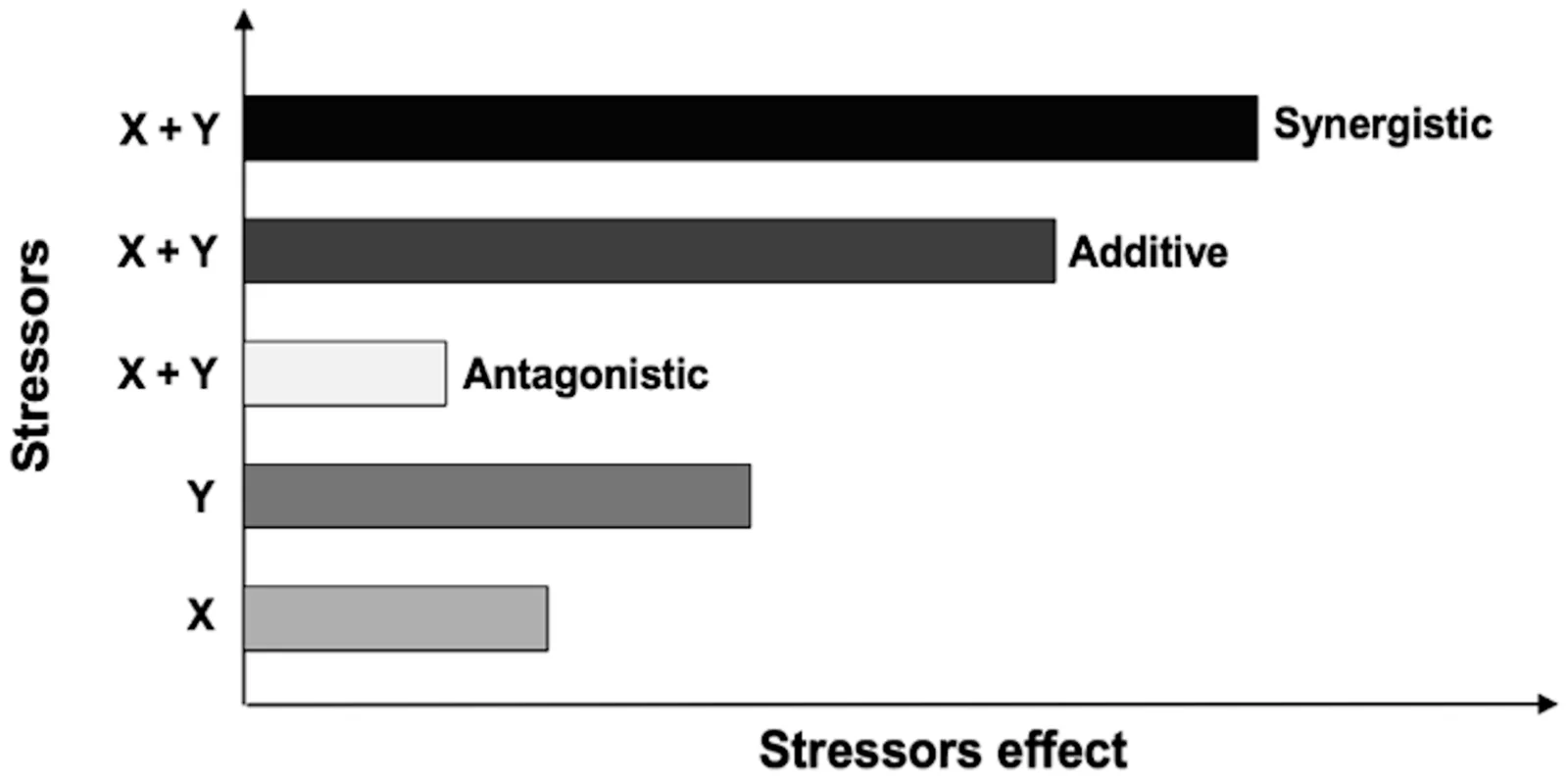
Types of stressors and their effects on animals’ physiological performance. Modified from Todgham and Stillman [11] and Gunderson et al. [12].

To classify interactions between stressors, it is necessary to first establish the physiological effects of isolated stressors [47,48]. What defines a “stressful condition” for an animal species is its optimal range of environmental conditions and its plasticity and adaptability to deal with fluctuations. This means that a “stressful” condition for a specific species may be within the optimal range for another species. Usually, animals that live in more unstable environments, such as the intertidal zone, have a greater adaptability to deal with extreme conditions, and this can happen in the same species living in different environments [49]. Once the isolated effect of each important environmental variable has been established, we can try defining the physiological effects of different stressor interactions in three categories: additive, antagonistic and synergistic effects (Figure 1; Glossary).

**Figure 2:**
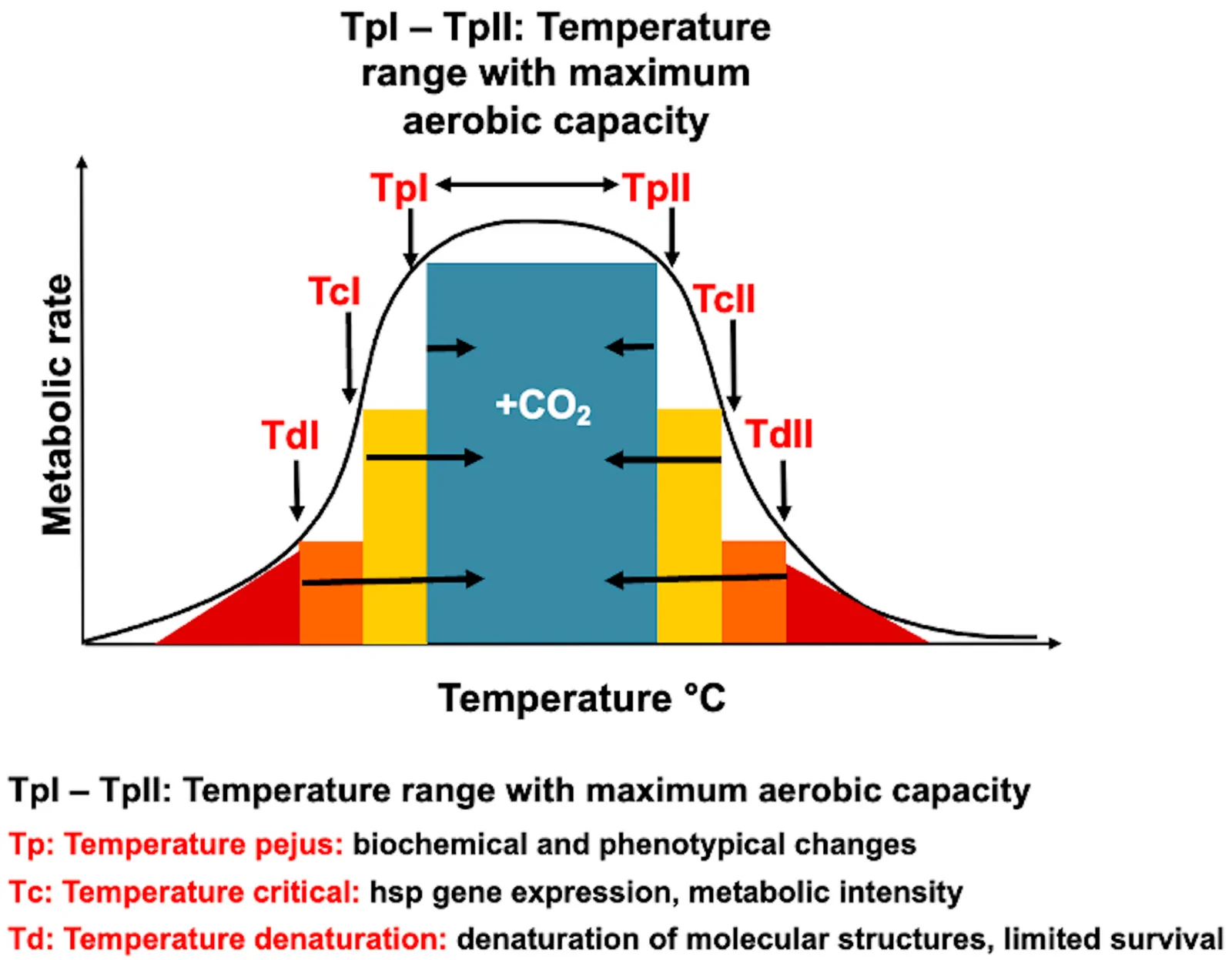
Narrowing of an organism’s thermal window under the effect of a second stressor; in this specific case, CO_2_ stands as the second stressor.

Examples of stressors’ antagonistic, additive, or synergistic effects have been widely documented in the literature. For example, a study examining the antagonistic effect has shown that increased exposure to ultraviolet radiation (UV-R; 280–400 nm) inhibited photosynthesis in the Antarctic macroalgae *Ulva bulbosa* (Hariot, 1889), which was ameliorated when increased temperature was simultaneously applied [50]. The additive effect has been shown in a study carried out by Anlauf et al. [51] in which *Porites panamensis* (Verrill, 1866) coral mass decreased by 45% when increased temperature was combined with low pH, by 21% when temperature was applied alone, and by 24% when low pH was solely applied. Examples of synergy have been given in both invertebrates and vertebrates. In the coral species *Acropora tenuis* (Dana, 1846), increased acidity decreased fertilization success by 7%, increased temperature by 15%, while the combination of both stressors decreased fertilization success by 39% [52]. The synergistic effect of increased temperature and elevated CO_2_ levels has been documented in the Mediterranean gilthead seabream *Sparus aurata* (Linnaeus, 1758) [53]. Specifically, while the sole effect of increased temperature constrains *S. aurata* aerobic and physiological performance [53,54], the addition of CO_2_ led to the narrowing of the species’ thermal window [53], according to the oxygen capacity-limited thermal tolerance (OCLTT) hypothesis [55] (Figure 2).

Despite the importance of studying and understanding stressor interactions, this classification framework has several limitations that make its universal application difficult. The intensity, duration, and order of stressor exposure determine whether interactions are additive, antagonistic, or synergistic [9,48]. Consequently, changes in one or more of these factors can lead to completely different physiological outcomes [47,48]. For example, a study on the tidepool sculpin (*Oligocottus maculosus*) demonstrated three different physiological responses of Hsps to temperature–salinity and temperature–hypoxia interactions: a +12 °C pretreatment had a synergistic effect, increasing tolerance to the second stressor (salinity or hypoxia), which was characterized as cross-protection; a 10 °C heat shock had no effect; and a +15 °C treatment had a deleterious effect [47]. From a biochemical perspective, physiological responses may also differ among tissues and analyzed parameters. Therefore, although this classification is important, it is also highly context-dependent and can easily change depending on the experimental design.

### 2.2. Cross-Protection

Cross-protection is a concept closely related to multistress, where exposure to a first stressor primes an organism to better withstand a second stressor (see Glossary; [7,8,9]). From a biochemical perspective, cross-tolerance occurs when a first exposure upregulates stress responses, such as antioxidant defenses and Hsps, increasing tolerance to subsequent stress exposure. The shared signaling pathways underlying these responses are referred to as cross-talk [8,9]. For example, ROS production activates transcription factors involved in the antioxidant system and the heat shock response [56,57]; both are crucial for environmental stress response. Due to the many research fields that study animals under multistress from different perspectives [4,5,6], the terms “cross-protection” and “cross-tolerance” can have different meanings. In this review, we use the definitions proposed by Rodgers et al. [8,9], in which cross-protection refers to the integrated phenomenon encompassing cross-tolerance and cross-talk (see Glossary).

The activation of cross-protection mechanisms depends on momentary factors, such as the duration, intensity, frequency, and order of stressor exposure, and chronic factors, such as sex and life stage [9]. It has been hypothesized that cross-protection evolved in environments where stressors occur in predictable and frequent sequences [8,9]. This means that, for an intertidal sessile animal, the activation of protective mechanisms may depend, for example, on whether UV radiation is intense enough during the aerial exposure caused by low tide, or whether UV is the first or second stressor [58,59]. For these reasons, studying and defining cross-protection phenomena in laboratory experiments is challenging, although they are important for elucidating the isolated physiological effects of individual stressors. Even minor experimental design choices determine whether cross-protection phenomena are detected or not. On the other hand, field studies capture physiological responses under realistic environmental conditions, although it becomes difficult to determine which stressor had more influence on the observed response.

The advantages and limitations of these two approaches are mainly related to cause-and-effect relationships [10]. For this reason, the best approach to investigate cross-protection phenomena is to integrate laboratory and field experiments to analyze isolated and integrated stressor responses. Previous reviews have discussed cross-protection phenomena in laboratory studies, analyzing more than one stressor simultaneously [7,9]; however, none have discussed how to observe this kind of response in nature, which is the most reliable approach. In this review, we discuss the multistress concept from a biochemical perspective, focusing on redox metabolism and heat shock proteins, eliciting this new concept in ecophysiology and describing the field approach to observe cross-protection phenomena. In Section 3 we discuss the field/natural experiment approach in multistress studies.

## 3. The Multistress Approach in Natural and Field Experiments

Field studies examining how environmental factors influence physiological traits typically involve conducting direct observations and collecting data on physiological responses and behaviors, and monitoring the environmental variables of interest in natural settings. To better understand the relationship between biochemical responses and environmental factors, it is essential to record as many variables as possible, such as temperature, ultraviolet radiation (UV-R), and other relevant conditions. The advantage of natural experiments is the ability to reveal how organisms respond under real-world conditions, encompassing the complexity and unpredictability of their natural environment [10,11,60,61].

Field studies can range from short-term to long-term projects, and from molecular analyses of physiological responses to broader population-level studies of adaptation and resilience. Successful field research requires careful planning, preparation, and execution to ensure the data collected is both accurate and meaningful in understanding the interplay between physiology and environment [12,62,63]. The unpredictability of natural conditions is the reality, and the laboratory experimental approaches do not always allow replicating and controlling the large number of biotic and abiotic stressors that occur in the natural environment [64]. Field studies can have minimal interference in environmental variables (field experiment) and no interference (natural experiment) by the researcher (see Glossary) [64]. Some studies employing the natural experiment approach in intertidal zones have identified time windows of interest for collecting animals that may have more pronounced biochemical responses [58,59,65,66]. Aquatic animals have been the subject of many studies with the natural experiment approach, including sea urchins (*Echinometra lucunter*), mussels (*Brachidontes solisianus*), gastropods (*Littorina sitkana*), and freshwater fish (*Astyanax elachylepis*) [58,59,65,66,67]. All these studies analyzed the effects of stressors related to the variations in oxygen concentration and/or availability in the natural environment in combination with other potential stressors, like temperature and salinity. They concluded that lack of oxygen was the main stressor driving the animals’ antioxidant response. However, in some studies [58,66], the recording of a broader range of environmental variables allowed for more detailed correlations between biochemical responses and environmental fluctuations. For example, in a sessile intertidal species, UV-R exposure during low tide may determine whether the antioxidant response is pronounced or not [58], as may the intensity of temperature fluctuation during emersion [66]. This shows the importance of recording environmental conditions at multiple time-points during sampling, as this allows for more precise correlations between biochemical responses and the fluctuating stressors in natural habitats.

Another approach is field experiments, which share many similarities with natural experiments but involve controlling one or a few variables to avoid unwanted dramatic effects that make the field study too invasive [10]. Researchers can erect a fence or cage to isolate animals, protecting them from predation while still exposing them to natural environmental conditions during sampling [17,68]. For example, in a field experiment conducted on the intertidal mussel *Perumytilus purpuratus* [68], researchers used corrals anchored to rocks to isolate individuals at different intertidal zones (upper and lower). Other examples of experimental manipulations in the field include shading or altering substrate color to perturb temperature, or filters to control UV radiation [17,69].

In contrast to laboratory experiments, natural and field experiments have become relevant nowadays due to environmental variability across different time scales (daily, seasonal, and interannual), providing a more realistic understanding of how abiotic and biotic factors influence animal physiology and behavior in nature. Each multistress study must consider the variables that are both relevant and influential for the species being studied, with those fluctuating at shorter time scales (e.g., diurnally) typically imposing greater physiological burden than changes occurring at larger temporal scales, which allow for physiological adaptation. For instance, in intertidal zones, fluctuations in temperature and ultraviolet radiation (UV-R) are critical factors, while salinity often changes on a more seasonal scale and is less immediately relevant [23]. However, in estuarine environments, salinity becomes a crucial variable, which must be considered due to its pronounced and frequent fluctuations [70]. Similarly, in mangroves, salinity fluctuations are also critical due to the mixing of freshwater and seawater, combined with low oxygen availability during tidal cycles and sediment composition [71]. Variables such as solar UV-R, on the other hand, are less impactful due to the dense vegetation canopy [65]. The relative importance of individual stressors varies across environments, and their combined effects must be interpreted within the ecological context in which organisms live. Therefore, the concept of multistress in each study depends on the natural habitat, the behavioral patterns, and the physiological characteristics of the species under investigation, as well as the time scale.

Aquatic organisms are exposed to a wide range of environmental variables that may act as stressors, either individually or in combination. Among these, fluctuations in salinity, oxygenation (hypoxia and hyperoxia), temperature, solar radiation, and pollutants represent some of the most influential factors shaping physiological responses in aquatic species. The following subsections focus on these key environmental stressors and their roles in shaping physiological and redox responses in aquatic animals. Evidence from various natural and field-based multistressor experiments is summarized in Table 1, detailing evaluated stressors. These in situ and environmental studies encompass aquatic organisms across several phyla, including Cnidaria, Annelida, Mollusca, Arthropoda, Echinodermata, and Chordata, spanning diverse habitats such as marine intertidal zones, estuaries, water columns, tidal pools, tropical lagoons, and water reservoirs.

### 3.1. Salinity

Environmental salinity is a key abiotic factor that can profoundly affect physiology and cellular responses, particularly in sessile marine invertebrates inhabiting coastal and estuarine ecosystems, where fluctuations can be abrupt and recurrent. Due to their inability to move, sessile organisms must regulate osmotic balance to maintain internal homeostasis, which not only requires the activation of active transport mechanisms but also leads to increased energy expenditure and cellular stress [72]. Simultaneously, salinity fluctuations compromise mitochondrial integrity, leading to energetic dysfunction in tissues with high metabolic demand [73]. Previous multifactorial studies in estuarine or mangrove systems (Table 1) have included monitoring of ambient salinity, as this factor is known to cause oxidative damage through changes in tissue ion concentrations, which have a high metabolic cost [72]. For example, it has been observed that the estuarine clam *Solen regularis* has a better ability to regulate its hemolymph osmolality, especially under hyperosmotic conditions (>25 psu), mainly due to a higher synthesis of SOD, CAT and GPx compared to hypo-osmotic conditions (<15 psu) [74]. For many estuarine organisms, intolerance to low salinities (<22 psu) means isolation from the external environment by valve closure (e.g., *Ostrea chilensis*), or by pedal muscle retraction against the substrate (e.g., the limpet *Crepipatella dilatata*) [24,75], which eventually leads to anaerobic metabolism activation and lactate accumulation [76]. However, it is the return to tolerable estuary salinity (>20 psu) which triggers high levels of oxidative damage and protein carbonyl levels, since the animal reconnects with the environment by shell opening, thus allowing the replacement of pallial cavity anoxic water by high-oxygen-content water with 7.0 mg O_2_ L^−1^ [19].

Although osmoconformation allows marine and estuarine organisms to maintain an isotonic balance with the surrounding environment, some organisms such as mussels, oysters, and clams have narrow tolerance ranges to salinity fluctuations (stenohaline). Therefore, changes in salinity levels beyond the limits of physiological tolerance, like those observed previously, lead them to isolate themselves from the surrounding environment, minimizing osmotic damage [77]. While this protects soft tissues from changes in ion levels, isolating from the surrounding environment also means that the microenvironment generated inside the pallial cavity will soon become an anoxic environment. Thus, reconnection with the surrounding environment during the high-tide period will generate ROS overproduction due to the shift from anaerobic to aerobic metabolism [19], a situation that requires these animals to prepare for oxidative stress (POS) as a strategy to minimize levels of oxidative damage. The above confirms that salinity is a factor that can not only cause high levels of oxidative damage directly, but moreover, reduced salinity levels can lead to the destruction of soft tissues. However, this damage can be prevented by isolation from the external environment, which results in a deprivation of available oxygen levels and, in turn, triggers a new problem: anoxia followed by reoxygenation.

### 3.2. Environmental Radiation

Environmental UV-R comprises different bands of sun electromagnetic radiation that impact the Earth’s surface, penetrating different aquatic ecosystems. Environmental radiation can be categorized into ultraviolet B radiation (UV-B; >280–320 nm), ultraviolet A radiation (UV-A; >320–400 nm), and photosynthetic available radiation (PAR; >400–700 nm) [78]. Due to its noxious effects inducing photo-oxidative damage, UV-B has been extensively studied [79], particularly in aquatic organisms that lack physical protection such as echinoderm larvae [17,18], anemones [15,23,80,81], corals [13,16], planarians [82], cephalopods [83] and gastropods [69]. For example, the seasonal photo-oxidative response of the symbiotic coral *Pocillopora capitata* in La Boquita Reef—Mexico exhibited elevated lipid peroxidation (TBARS) during summer when UV-R, water temperature and chlorophyll reach their maximum levels of the year [13]. In the same context, a field-multifactorial (salinity, temperature, UV-B, UV-A and PAR radiation) experiment, carried out in the Quempillén estuary, indicated that salinity fluctuation by tidal changes did not significantly affect oxidative damage levels in the symbiotic sea anemone *Anthopleura hermaphroditica*, although UV-R decreased its photosynthetic performance (F_v_/F_m_, ETR_max_ and E_k_) [84].

Similarly, Lister et al. [17] showed that UV-B radiation generated elevated levels of oxidative damage and deformities in larvae of the sea urchin *Tripneustes gratilla* when compared to those exposed only to PAR levels. Daily variations in radiation parameters such as those mentioned above can be exacerbated by seasonality, since the transition from winter to summer increases UV-B doses by 23 percent in a mid-latitude area of the southern hemisphere (New Zealand) [80]. In compliance with the aforementioned, a field study conducted in the Quempillén estuary showed that seasonal changes between winter and summer can cause increases in temperature levels, UV-B radiation dose (280–320 nm) and PAR radiation (400–700 nm) by 2-, 10- and 6-fold, respectively, resulting in a 4- and 20-fold increase in lipid peroxidation and protein carbonyl levels in *A. hermaphroditica* [23].

Most multifactorial natural and field experiments that assessed the impact of UV-R in aquatic invertebrates (Table 1) have considered the estimation of at least UV-B radiation through a daily cycle; however, depending on the research question, environmental PAR must be considered, especially if the biological model is a symbiotic complex (e.g., anemone, corals, sea slugs, mollusks) that uses PAR light to photosynthesize. Under conditions of high PAR irradiance, the overexcitation of photosynthetic centers—as well as mitochondrial and chloroplast electron transport systems in photosynthetic organisms—can lead to electron leakage toward molecular oxygen, resulting in the formation of ROS and subsequent oxidative damage. A recent study on the symbiotic nudibranchs *Elysia velutinus* and *Elysia ornata* (which rely on kleptoplasty—chloroplasts sequestered from algae) demonstrated that elevated PAR levels induced a significant increase in oxidative damage. Nevertheless, these organisms escape towards shaded areas or accumulate chloroplasts in specific body regions to act as a defensive strategy against excess PAR [85]. In macroalgae, the response to high PAR exposure includes dynamic photoinhibition processes, characterized by a transient reduction in photosynthetic efficiency and thermal energy dissipation, aimed at minimizing oxidative damage [86].

It is essential to consider the temporal cyclicity of solar radiation when designing natural and field experiments aimed at evaluating its cellular or physiological impact on animals. Solar irradiance varies across both daily and seasonal scales, significantly modulating the magnitude of photo-oxidative stress experienced by organisms. On a daily scale, irradiance typically peaks around noon, coinciding with the highest levels of photo-oxidative damage [23,87], while seasonal patterns shape both the intensity and duration of light stress throughout the year, resulting in variable levels of oxidative stress [80] (Table 1). A frequently underestimated factor in such field research is the influence of transient atmospheric conditions, particularly the presence of broken clouds, which can temporarily enhance irradiance levels and amplify photo-oxidative effects [88,89,90]; light scattering at the edges of clouds transiently increases both direct and diffuse solar radiation reaching the surface. This effect can trigger acute increases in cellular damage, occasionally surpassing the levels observed on fully clear days [89].

### 3.3. Temperature

Temperature is a central factor in multistressor studies due to its overarching influence on all physiological, biochemical, and cellular processes in ectothermic organisms [68]. Thermal fluctuations can alter metabolic rate, affect membrane fluidity, modify enzyme kinetics, and destabilize redox balance, thereby modulating both vulnerability and the physiological capacity to respond to other stressors such as hypoxia, salinity, acidification, or solar radiation [91]. In the context of combined stress, temperature functions not only as a direct stressor but also as a modulator that can amplify or mitigate the effects of co-occurring factors depending on the intensity, duration, and sequence of exposure. For instance, elevated temperatures may enhance ROS production under hypoxic conditions or increase susceptibility to osmotic damage during salinity shifts [92,93]. Therefore, incorporating realistic thermal gradients into multistressor experimental designs is essential for understanding the true tolerance capacities and physiological thresholds of organisms under climate change scenarios. Undoubtedly, temperature levels fluctuate in both daily cycles and broader seasonal patterns (Table 1). For example, the impact of seasonality on oxidative response in the estuarine anemone *A. hermaphroditica* in the Quempillén river (39° S) during the austral summer and winter seasons indicated that during summer, elevated levels of lipid peroxidation are results of increased temperature and ultraviolet B radiation, while during winter, tidal reductions during midday generate the main levels of damage to lipid membranes due to salinity reductions in the estuary because of continuous precipitation [23].

While temperature is an abiotic factor related to UV-R radiation, the simultaneous presence of both is not implicit. The temperature that reaches the Earth’s surface comes from infrared radiation emitted by the sun, which impacts organisms either directly or indirectly by increasing the rate of enzymatic reactions and generating high levels of oxidative damage when physiological tolerance ranges are exceeded. Thus, stochastic fluctuations in natural environments, such as marine heat waves (MHWs) in coastal areas, can generate drastic changes in oxidative damage levels. This confirms that temperature is the major metabolic modulator that can generate elevated levels of oxidative damage in marine organisms.

**Table 1:** Oxidative damage studies in aquatic animals based on the evaluation of multiple stressors in nature experiments.

Phylum/Class	Species/Environment	Environmental Stressors	Reference(s)
Cnidaria Anthozoa	*Anemonia viridis* Intertidal pools Field survey	water temperature salinity pH	[94]
	*Anthopleura hermaphroditica* Estuarine tidal change	water temperature salinity UV-B radiation UV-A radiation photosynthetically active radiation temporal–seasonal study	[23]
	*Pocillopora capitata* Field survey La Boquita Reef	surface water temperature water column temperature photosynthetically active radiation temporal–seasonal study	[13]
Annelida Polychaeta	*Sabella spallanzanii* Field survey Subtidal sediment	metal pollution (Cd, Cr, Cu, Fe, Mn, Ni, Pb, and Zn)	[95]
	*Laeonereis acuta* Field survey Estuary	temporal–seasonal study temperature salinity pH pollution degree (polluted/unpolluted)	[96]
Mollusca Bivalvia	*Perumytilus purpuratus* Transplant experiment Intertidal rocky shore (low tide 12:00–14:00)	UV-B radiation air/water temperature rock temperature shell temperature tissue temperature relative humidity bathymetric intertidal position (upper/lower)	[68]
	*Mytilus californianus* Transplant experiment Intertidal rocky shore	temperature seasonal acclimation (may/august) bathymetric intertidal position (upper/lower)	[97]
	*Brachidontes solisianus* Intertidal rocky shore (low tide 12:00–14:00)	air/water temperature aerial exposure bathymetric position (emerged/submerged)	[59]
	*Brachidontes solisianus* Intertidal rocky shore (low tide 12:00–14:00)	temperature salinity UV-R bathymetric position (emerged/submerged)	[58]
Mollusca Bivalvia	*Mytilus edulis* *Estuary*	water temperature salinity total suspended solids turbidity chlorophyll a pheo-pigments phycotoxins	[20]
Mollusca Gastropoda	*Tegula bunnea* *Tegula funebralis* Intertidal rocky shore Transplant experiment	temperature tidal height sun exposure degree	[98]
Mollusca Polyplacophora	*Chaetopleura angulata*	air temperature water temperature salinity water pH temporal–seasonal study (spring and summer)	[99]
Arthropoda Crustacea	*Palaemon elegans* Tidal rock pool	air temperature water temperature salinity water pH temporal–seasonal study	[100]
	*Ucides cordatus* Estuary	salinity conductivity temperature pH dissolved oxygen seasonal (dry–transitional–rainfall) estuary position (mouth, middle, and head) biometry (total length, width, and height)	[101]
	*Tripneustes gratilla* Tropical lagoon	UV-B radiation UV-A radiation photosynthetically active radiation sub-superficial level (shallow/depth)	[17]
Echinodermata Echinoidea	*Sterechinus neumayeri* Antarctica	UV-B radiation UV-A radiation PAR radiation atmospheric ozone concentrations (DU) ice cover/open ocean	[18]
	*Echinometra lucunter* Intertidal zone Field survey	air/water temperature solar radiation dissolved oxygen	[66]
	*Sparus aurata* Water column	water temperature (at each depth) water pH salinity oxygen temporal study (monthly)	[102]
Chordata Pisces	*Lipophrys trigloides* Tidal rock pool (low tide 12:00–14:00)	air temperature water temperature water pH salinity	[103]
	*Astyanax altiparanae* *Freshwater reservoir*	dissolved nutrients metal concentration (Al, Ar, Ba, Cd, Cr, Co, Cu, Mn, Ni, Pb, Sr, and Zn) chlorophyll pharmaceutical drugs temporal study (seasonal) internal organs (gills/liver)	[25]
	*Sciades herzbergii* *Water column* *Estuary*	temperature pH salinity dissolved oxygen (DO) seasonal study bottom sediment samples trace elements (Al, Cd, Cr, Cu, Fe, Mn, Ni, Pb, and Zn) total mercury	[104]

### 3.4. Oxygen Availability (In Laboratory and Field)

Hypoxia is particularly relevant in aquatic systems where oxygen availability fluctuates on a seasonal or daily scale. Field studies allow researchers to investigate how hypoxia interacts with other abiotic factors to impact redox balance, as variations in oxygen availability often co-occur with changes in other environmental factors such as temperature, UV radiation, and pH, which can interact and exacerbate hypoxia. For example, in many coastal and intertidal ecosystems, oxygen levels rise during the day due to photosynthesis, sometimes reaching hyperoxic levels, and decline at night due to respiration, potentially causing hypoxia [105,106] and exposing many intertidal species to hypoxia–reoxygenation cycles [107,108,109]. In these environments, increased temperatures (which may also be associated with increased UV radiation) can reduce dissolved oxygen levels while also stimulating increased metabolic demand of aquatic species [110,111].

In intertidal zones, hypoxia also occurs naturally during emersion (air exposure) at low tide, leading to what is called functional hypoxia. As sessile organisms are unable to migrate, they are distributed along the coast according to their physiological tolerance to abrupt fluctuations in temperature and UV radiation, which are intensified during air exposure, a phenomenon known as vertical zonation [112]. To survive these extreme environmental shifts, these animals have evolved integrated biochemical strategies, with redox metabolism serving as a primary line of defense to mitigate oxidative damage caused by the reoxygenation during tide return [58,65,66].

While hypoxia occurs naturally in many systems, anthropogenic drivers (e.g., pollution, increased nutrient input) have contributed to hypoxia occurring outside of regions or intensities that it has historically, contributing to the expansion of dead zones and eutrophication [113,114]. Hypoxia driven by eutrophication is now recognized as the chief stressor in marine ecosystems, capable of causing mass mortality events and occurring across seasonal, episodic, or persistent temporal scales [115,116].

Hypoxia and reoxygenation are known to alter redox balance by promoting ROS production, antioxidant, and mitochondrial defense mechanisms [117,118,119,120,121]. Furthermore, there is a large body of literature highlighting how redox responses to hypoxia are highly modulated by temperature, pH, and salinity [92,122,123]. For example, pearl oysters (*Pteria penguin*) that were subjected to different durations of air exposure combined with temperature variation showed a rapid increase in SOD, CAT, TAOC, and GSH-Px activities in response to short-term air exposure, indicating an adaptive response (cross-protection). However, when maintained for 12 h at 30 °C, survival dropped below 20%, indicating a deleterious effect of increased temperatures [124]. In the case of *C. gigas*, when acclimated to low salinity, mitochondrial performance was improved following hypoxia and reoxygenation relative to those exposed to high salinity, but there were higher levels of mitochondrial ROS efflux [125]. The mussel *Mytilus coruscus* exhibits antioxidant responses to low oxygen availability and decreased pH, and the effect of these two stressors together was additive, with SOD, CAT, and ACP levels being highest when both stressors occurred [122].

Taken together, these studies demonstrate how responses to hypoxia are highly modulated by additional stressors. While some species may be well adapted for hypoxia alone, the consequences of naturalistic hypoxia cannot be fully understood in isolation from other common environmental stressors that often co-occur with hypoxia. Therefore, field and natural studies are important for capturing a more realistic picture of how animals cope with hypoxia under multistress conditions, and the great level of diversity across taxa and their physiological responses to such stressors [92,107,117,118,122,126,127,128,129].

## 4. Animal Stress and Heat Shock Proteins

### 4.1. General Information on Hsps

Since the discovery of heat shock proteins (Hsps) in the salivary glands of the *Drosophila* larvae in 1962 by Ferruccio Ritossa [130], constant research regarding their important functions has cemented their key role in animal cells as a common molecular stress response to internal and external stressful signals [131,132]. Although under unstressed conditions, Hsps perform housekeeping duties and regulation of protein quality control, versatile stressors such as temperature, pH, salinity, etc. [133,134,135,136] can lead to protein denaturation, degradation of misfolded proteins, and protein aggregation, which can in turn induce Hsp production [137], setting them as key indicators of environmental stress [130,138], which negatively impacts the overall fitness of animals [139,140,141,142]. Although the precise mechanisms through which environmental, abiotic and biotic stressors induce Hsp production are not fully understood in animals, it is widely accepted that they greatly disturb cellular homeostasis, thus altering physiological processes such as growth, reproduction and survival [143]. Their cell-protective role consists of proper protein folding and translocation of organelles across cellular membranes [136,137,144]. Hsp induction has been proposed as a potential link that can modulate homeostasis in response to environmental stress, enabling organisms to adapt and survive. While the common signal generated by various stress stimuli is likely to be protein damage, the operation of the cellular stress sensing mechanism is not yet fully known [145,146].

**Figure 3:**
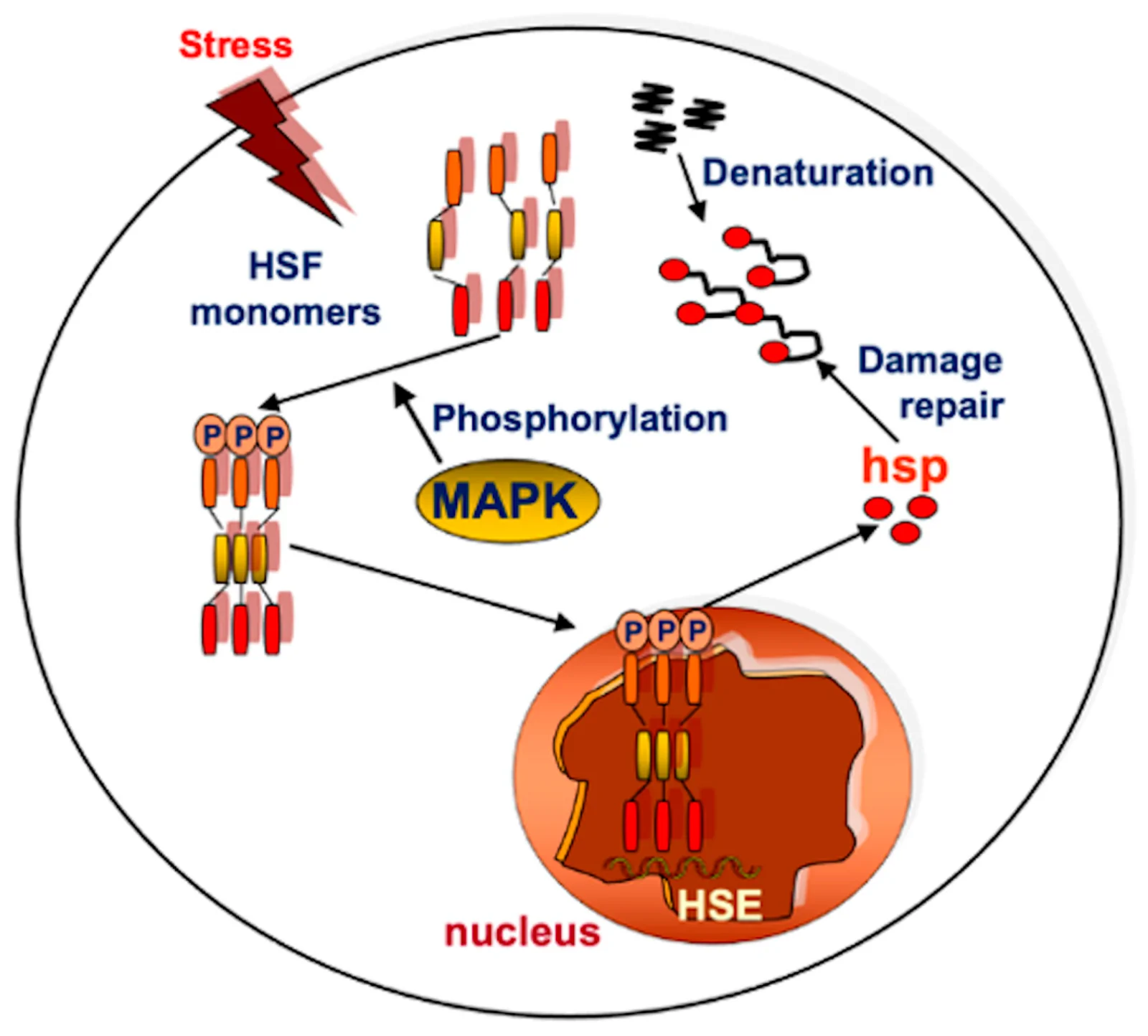
Transcriptional activation of Hsps by the translocation and trimerization of HSF (P: phosphorus group, MAPK: mitogen-activated protein kinase, HSF: heat shock factor, HSE: heat shock element, hsp: heat shock protein).

The inducible Hsp expression is regulated by the heat shock transcription factors (HSFs). Specifically, the effect of various stressors (e.g., elevated temperature, hypoxia, salinity fluctuations, acidification, pollutants, and oxidative stress) leads to the acquisition of HSFs’ DNA binding activity to the heat shock element (HSE), a domain of the promoter region of heat shock genes, which results in Hsp production and cellular accumulation [56,57,147]. Specifically, when HSF receives reversible phosphorylation by mitogen-activated protein kinases (MAPKs) [148,149,150,151,152], it is translocated into the nucleus where it trimerizes [153] and binds to the HSE [154], leading to Hsp transcriptional activation (Figure 3). However, in unstressed cells, due to the presence and the negative regulation of Hsps by interacting with the oligomerization domains (HR), HSF1 exists in a monomeric state [147,155,156,157]. With an increase in ROS levels, SH groups on the surface of Hsp70 and Hsp90 are oxidized, and as a result Hsps are dissociated from HSF1 [154] and released from the HSF1 complex, followed by HSF1 trimerization and the transcriptional activation of Hsps [147]. Consequently, HSF1 functions as a central integrator of diverse environmental stress signals that converge on proteome instability. Since the isolation of a single HSF gene from *Saccharomyces cerevisiae* [158,159] and *Drosophila melanogaster* (Meigen, 1830) [160], several HSF members have been found in vertebrates (HSF1, HSF2, HSF3, HSF4), which suggests that different HSFs mediate the responses to various forms of physiological and environmental stimuli [161].

Although Hsp induction is generally considered a protective response that enhances cellular homeostasis and stress tolerance, its activation is associated with significant energetic costs. Specifically, the synthesis of Hsps, together with their ATP-dependent chaperone functions (protein folding, refolding, and degradation) can substantially increase energy demands. Under stressful conditions where ATP production may already be constrained, the maintenance of heat shock response may result in physiological trade-offs affecting growth, reproduction, immune function, and other energy-demanding processes. Therefore, the benefits of Hsp induction should be considered in the context of the energetic capacity of the organism and the duration and intensity of stress exposure [162,163].

This complex network of molecular chaperones is primarily grouped into two major groups according to their amino acid composition, molecular weight, and specific cellular function: the high- and low-molecular-weight Hsps (60 to 110 kDa and 15 to 43 kDa) [131,132,164]. While both groups are ATP-dependent, their cellular function is different (Table 2):

- High-molecular-weight Hsp functions are binding and folding of nascent proteins through ATP-dependent allosteric organization, which consist of assembling, transportation, and degradation of improperly folded peptides, but they also play critical roles in cell signaling, immune response modulation, and preventing apoptosis, thus promoting cell survival under stress, which is crucial for maintaining homeostasis [165,166].
- Low-molecular-weight Hsp functions have been documented in ATP-independent molecular chaperoning, acting as early responders to cellular stress by binding damaged or unfolded proteins and preventing their irreversible aggregation. They preserve proteostasis, reduce toxicity, and support cell survival [131,136,167].

**Table 2:** Classification of Hsp family members and their role.

Hsp Member	Cellular Function	Cellular Location	Reference(s)
Hsp110	Protein refolding with Hsp70 Cell survival under stress	Cytoplasm Nucleus	[168]
Hsp100	Refolding of aggregated or misfolded proteins	Cytoplasm	[169]
Hsp90	Signal transduction Sarcomere formation Myosin folding	Cytoplasm	[136,170]
Hsp70	Protein assembling Protein folding Degradation of improperly folded proteins Translocation of organelles	Cytoplasm Nucleus	[136]
Hsp60	Protein folding Prevention of protein aggregation Prevention of assembling of unfolding proteins	Cytoplasm Mitochondria	[171]
Hsp40	Assistance in protein folding with Hsp70	Cytoplasm	[172]
Hsp27	Refolding of denatured proteins	Cytoplasm Endoplasmic reticulum Nucleus	[173]
Hsp10	Assistance in protein folding	Mitochondria	[174]

### 4.2. Multistressors’ Effect on HSPs: Laboratory Experiments

While several studies in the field of ecophysiology have focused on the effect of single stress factors as well as their combination on invertebrates’ and vertebrates’ physiological performance, only a few of them have involved the study of the heat shock response (HSR) as a potential stress indicator. Nonetheless, the importance of Hsps in the maintenance of protein homeostasis, their response to various stressors, their involvement in cellular pathways (e.g., signaling transduction, cell cycle, and apoptosis regulation) and their role in diseases such as cancer and neurodegeneration, is currently highlighted (e.g., [175,176,177]). Research underlining the ecophysiological significance of these molecules can be found below (Table 3).

Regarding invertebrate species, multistress effects have been examined in zooplankton, bivalves, and amphipods in many laboratory simulations. Specifically, since the keystone grazer *Daphnia magna* (Straus, 1820) has faced dramatic water chemistry changes in its environment over five decades, temperature as a single stressor and in combination with food levels and insecticide loads has resulted in both a downregulation and an upregulation of Hsps after a temperature ramping treatment in the presence of multiple stressors. The latter has been attributed to a high degree of plasticity because of diverse stressors affecting HSP expressions in different directions [178].

Marine ectotherms such as eastern oysters (*Crassostrea virginica*) (Gmelin, 1791) are also commonly exposed to multiple environmental stressors, such as temperature and heavy metal pollution. The combined effect of these stressors (acute temperature stress and/or long-term Cd exposure) on this oyster species has shown that the exposure to both stressors simultaneously generates a suppression of Hsp levels compared to the effect generated by one stressor alone, thus indicating a decreased thermotolerance of oysters from metal-polluted environments [179]. Increasing temperatures can be significantly stressful for all aquatic organisms, including amphipods, which represent the most abundant and functionally important groups of freshwater macroinvertebrates. Exposure of *Gammarus pulex* (Linnaeus, 1758) to a 10 °C temperature increase (from 15 °C to 25 °C) and microsporidian infections caused an increase in Hsp70 protein level, in addition to other stressors, suggesting that such an increase might have a protective effect for the host against acute temperature stress [180].

Among the other ectothermic organisms in which the multistress effect has been investigated are vertebrates, with a predominant focus on fish. In this context, exposure of the economically important fish species *S. aurata* to increased temperatures of 24 °C and 28 °C (compared to control of 18 °C) and decreased pH levels (due to increased CO_2_ levels dissolved in the water) led to an exacerbation of the hypoxemic conditions caused only by one stressor, as also evidenced by the persistent expression of Hsp70 and Hsp90 [53]. Similarly, the effect of dual stressors on the shark catfish species *Pangasinodon hypophthalmus* (Sauvage, 1878) has also been examined. Specifically, fish reared under lead (Pb 4 ppm) and high-temperature (34 °C) stresses, fed with dietary selenium nanoparticles (Se-NPs, 1 mg/kg, 2 mg/kg) or without Se-NPs (0 mg/kg), exhibited devastating impacts on fish health status and increased stress indicators, such as Hsps, compared to those reared under one stressor effect. However, dietary Se-NPs exhibited a capability to enhance overall performance and alleviate multiple stresses in *P. hypophthalmus* [181]. While one of the two stressors regarding the aforementioned studies is temperature, in the Chinook salmon *Oncorhynchus tshawytscha* (Walbaum, 1792), the effect of the pyrethroid pesticide esfenvalerate and the organophosphate pesticide chlorpyrifos, as well as virus exposure, was examined. The highest Hsp levels were observed after the combined treatments, suggesting an additive effect between virus and pesticides [182].

Collectively, the available evidence highlights the ecophysiological relevance of Hsps as sensitive biomarkers of exposure to multiple stressors, reflecting cellular attempts to maintain proteostasis under adverse conditions. Variability in Hsp expression across taxa and stressor combinations likely arises from differential activation of stress-sensing pathways, including HSF-mediated transcriptional regulation, redox signaling, and energy-dependent chaperone activity involved in protein folding, refolding, and degradation. These molecular responses can promote cytoprotection by limiting protein misfolding and aggregation, but under prolonged or severe stress may also reveal energetic constraints and dysregulation of cellular homeostasis, ultimately leading to maladaptive outcomes. Further integrative studies combining molecular, physiological, and ecological approaches are needed to elucidate how stress-response networks shape organismal resilience under the combined pressures of global environmental change.

**Table 3:** Expression of Hsp families from different species under multistress effects.

Species	Stressor	Stress Duration	Stressor Effect	Hsp Member	Reference(s)
*D. magna*	temperature insecticide loads	24 and 48 h	additive antagonistic	Hsp20 Hsp60 Hsp70 Hsp90	[178]
*C. virginica*	temperature heavy metals	1 h	antagonistic	Hsp70 Hsp90	[179]
*G. pulex*	temperature microsporidian infections	24 h	antagonistic	Hsp70	[180]
*S. aurata*	temperature CO_2_	1, 3, 5 and 10 days	synergistic	Hsp70 Hsp90	[53]
*P. hypopthalmus*	temperature heavy metals	60 days	additive	Hsp70	[181]
*O. tshawytscha*	pesticides viral infections	60 days	additive	Hsp60 Hsp70 Hsp90	[182]

### 4.3. Multistressors’ Effect on HSPs: Natural and Field Experiments

Although the importance of Hsps has been previously largely discussed in laboratory-oriented studies, they are also highlighted as powerful tools for the definition of extreme thermal limits in research regarding the effect of natural environmental conditions on the biology and physiology of several animal species, which vary from invertebrates such as bivalves, snails and crustaceans to vertebrates such as fish and amphibia (Table 4). Considering that numerous laboratory studies have demonstrated the ability of species to initiate a cellular stress response under harsh environmental conditions, the aim of this section is to underline the relationship between these conditions and heat shock response (HSR). Environmental monitoring and management must account for these effects to avoid the interference of seasonal confounding variables. However, Hsp expression remains highly versatile; it is modulated by tissue type, the intensity and duration of the thermal stress, and the specific thermal history of each species [183,184].

In the subtidal horse mussel, *Modiolus modiolus* (Linnaeus, 1758), seasonal collections from a depth of 10 m at the Isles of Shoals, New Hampshire, revealed that the concentration of Hsp70 was greater in winter than in summer samples, suggesting that protein chaperone functions must be maintained while other seasonal adjustments to cold temperatures are occurring, such as an overall metabolic and antioxidant compensation [185]. The pattern of Hsp induction was different in the Mediterranean mussel *Mytilus galloprovincialis* (Lamarck, 1819), where the effect of temperature was clear and the pattern of response was biphasic in the posterior adductor muscle and mantle: Hsp72 and Hsp90 increased between February and April, remained constant until the end of May, and thereafter gradually increased towards elevating summer temperatures, from June to August, indicating increased protein turnover or protein damage [186]. In the intertidal mussel *Mytilus trossulus* (Gould, 1850), where animals were subjected to a wide range of body temperatures (from approximately 10 °C to 35 °C in summer), both summer-collected and intertidal mussels exhibited greater Hsp70 levels compared to winter-collected and subtidal individuals, respectively. These results show that high summer temperatures cause increased denaturation of cellular proteins in summer-acclimatized mussels, which persist despite the increases in the levels of heat shock proteins [187].

The land snail species *Helix lucorum* (Linnaeus, 1758) displays a wide altitudinal distribution, resulting in populations being exposed to different seasonal temperature variations. Individuals from two different populations, one at sea level (0 m) and the other at Olympus Mountain (1250 m), as well as after mutual population exchanges, showed seasonally altered Hsp70 and Hsp90 levels both among different populations and between the same populations exposed to varying altitudes. However, individuals of the same population in their native habitat or acclimatized to a different habitat showed a relatively similar pattern of expression, supporting the induction of the specific proteins according to the life history of each species [188]. Contrary to the aforementioned mollusk species, in the temperate shrimp species *Palaemon elegans* (Rathke, 1837), seasonality affected several biochemical factors in an extremely versatile way—shifting upwards, downwards, or remaining constant—thereby employing different biochemical strategies depending on the season: while catalase activity and lipid peroxidation increased in summer, consistently induced Hsp70 levels represent a mechanism of seasonal acclimatization involving the protein quality control system; this is particularly important for intertidal populations, which are frequently exposed to constantly changing environments with temperature having a major change in the seasonal comparison [189]. Beyond environmental temperature changes, organisms also cope with other variables which seasonally change such as UV-R. The zooplankton copepod from the clear alpine lake species *Cyclops abyssorum tatricus* (Kozminski, 1927) exhibited pronounced upregulation of *hsp70* gene expression that inversely correlates with its levels of photoprotection (e.g., mycosporine-like amino acids and carotenoids) in February during the ice-cover period, when photoprotective defenses are at their annual minimum. Despite the physical shielding provided by the ice against UV, these findings suggest that Hsp70 expression is modulated by seasonal plasticity—driven by both water temperature and the organism’s internal defensive state—ensuring survival in highly variable alpine ecosystems [190].

Another study focused on the mitochondrial Hsp70 (mtHsp70) levels in the liver mitochondria of the grey mullet *Mugil cephalus* (Linnaeus, 1758) collected from a polluted and an unpolluted site over the course of monsoon and summer. Fish exposed to chronic environmental stress in the polluted site overexpressed mtHsp70, p-HSF1 (nuclear fraction) and Hsp90α (together with higher lipid peroxidation), particularly during summer to cope with the increased seasonal and pollution stress, compared to the unpolluted area [191]. Contrary to the Hsp70 levels from the previous study and regardless of the pollution factor, both Hsp90α and p-HSF1 seasonally differed, with their levels increasing in the summer (increases were, however, higher in the polluted estuary) [192]. It seems, therefore, that Hsp90 responds differentially compared to Hsp70: it could be proposed to be a more sensitive indicator of stress in *M. cephalus* since it is also induced by seasonal environmental stress.

In a different fish species, *S. aurata*, Hsp induction and MAPK activation—which exhibited a seasonal pattern characterized by distinct tissue specificity—occurred before peak summer water temperatures, with no further increase despite subsequent rises in temperature. This activation, triggered by cold acclimation, could be interpreted as a preparatory strategy by the fish to cope with the high ambient seawater temperatures (beyond Tc) recorded during the summer [54]. Similar to other farmed species such as *S. aurata*, the seasonal patterns of physiological responses in the red porgy, *Pagrus pagrus* (Linnaeus, 1758), may support the concept of eurythermy. Particularly, during seasonal field acclimatization, both Hsp70 and Hsp90—as well as other stress indicators belonging to the apoptotic and autophagic pathways—exhibited high levels in parallel with the increasing seawater temperature in the spring. However, the increased levels of several bio-indicators during cold acclimatization suggest a complex biochemical response to changing field conditions [193].

A different strategy was employed by the amphibian *Pelophylax ridibundus* (Pallas, 1771) during an 8-month acclimatization period in the field (November–June). Specifically, in the heart and the gastrocnemius muscle of the water frog, the induction of Hsp70 and Hsp90 were retained at low levels due to a general metabolic depression during winter hibernation. Recovery from this phase induced an increase in the levels of both these proteins, underlining their cell-protective role and adaptation to environmental stress [194], probably as a preparation for oxidative stress (POS) strategy during arousal from overwintering [195,196].

The evaluation of the physiological and cellular responses of organisms under natural or semi-natural conditions requires a comprehensive understanding of how multiple environmental stressors interact across spatial and temporal scales. Unlike traditional laboratory experiments that often isolate single variables, field and natural experiments better capture the ecological complexity and unpredictability of the habitats in which these organisms live. Environmental parameters such as temperature, salinity, oxygen availability, and solar radiation—including UV-R and PAR—do not act independently; rather, they interact dynamically, often synergistically, producing biological effects that cannot be predicted from single-stressor studies. Furthermore, the temporal structure of environmental fluctuations, including diel and seasonal cycles, and the influence of events such as intermittent solar exposure or extreme weather events must be accounted for when assessing oxidative stress and other physiological endpoints. As shown across a wide range of species and ecosystems, realistic multistress approaches—grounded in robust environmental monitoring—are essential to uncovering the true thresholds of tolerance, the activation of “anticipatory” mechanisms like the POS response, and the adaptive strategies that underlie survival in ever-changing environments.

## 5. Animal Stress and Redox Metabolism

Aerobic life has evolved a sophisticated signaling system that relies on oxidation and reduction of biomolecular targets mediated by dynamic interactions among reactive species, oxidizable targets, neutralizing agents (antioxidant systems), and repair mechanisms in a reactive species interactome [197]. These interactions underpin redox signaling, in which reactive species act as regulated messengers essential for normal physiological functions rather than merely as damaging byproducts of metabolism. Such “redox code” integrates metabolism with cellular signaling [198], regulating a wide range of cellular processes, including cell proliferation, metabolism, gene expression, and immune response [199]. Thus, the redox metabolism is essential for maintaining normal cellular and physiological function.

### 5.1. Oxidative Stress and Antioxidants

Reactive oxygen species (ROS) are naturally produced during metabolism, particularly from the mitochondrial electron transport chain and other redox-active enzymatic systems, such as NADPH oxidases [200]. Cells rely on antioxidant systems composed of antioxidant enzymes (e.g., superoxide dismutases, catalase, glutathione peroxidases, peroxiredoxins), proteins (e.g., thioredoxin and metallothionein) and low-molecular-weight antioxidants (e.g., glutathione and ascorbate) to manage ROS levels and preserve redox signaling while preventing uncontrolled oxidation [201]. The functioning of antioxidant systems, such as glutathione- and thioredoxin-dependent systems, is greatly dependent on reducing equivalents in the form of NADPH, since NADPH is required for the activity of glutathione reductase and thioredoxin reductase to recycle the oxidized forms of glutathione and thioredoxin to their reduced states [202,203]. Given their reactivity, the oxidation of biomolecules by ROS cannot be entirely prevented, even under non-pathological conditions, despite the multi-layered antioxidant systems in place. Thus, cells employ several mechanisms to repair or replace oxidatively damaged biomolecules, including DNA repair pathways, reduction of oxidized residues in proteins, and protein degradation [204]. Here, again, NADPH is critical for supplying reducing equivalents for biosynthetic pathways to replace damaged molecules and also as a cofactor for repair enzymes [199], such as aldehyde reductase [205].

Redox metabolism operates in such a way that even under non-stress conditions some degree of oxidative modification of biomolecules occurs and it exerts its homeostatic function, a state defined as oxidative eustress [206]. For instance, quantitative redox-proteomics demonstrated that many protein cysteines are constitutively only partially reduced, indicating a substantial oxidized fraction under basal conditions [207], highlighting that controlled oxidation is not necessarily detrimental to cell function. Under certain conditions, however, such as exposure to an environmental stressor, the production of reactive species might surpass the managing capacity of antioxidant systems, resulting in excessive oxidative modification, often irreversible and causing loss of function, characterizing oxidative distress [206]. Regardless of the primary cause, disruptions of the redox balance trigger transcriptional responses, such as those mediated by the redox-sensitive Nrf2, FoXOs, and NF-κB transcription factors [208,209,210]. These ultimately upregulate genes encoding antioxidant enzymes, detoxification proteins, and NADPH-generating enzymes [196]. The products of these genes help to bring the redox balance back to its basal state of oxidative eustress. Experimentally, the redox metabolism is often investigated by determining the levels of antioxidant enzymes and redox-sensitive transcription factors at the transcript, protein or activity level; non-protein antioxidants; and oxidative stress markers, such as protein carbonyls and malondialdehyde as indicators of oxidative damage to proteins and lipids, respectively.

Among the transcription factors that regulate antioxidant gene expression in response to oxidative stress, the Nrf2/KEAP1/ARE axis is the most extensively characterized. Under basal conditions, Nrf2 is constitutively bound to its cytosolic repressor KEAP1, which acts as a substrate adaptor for a ubiquitin ligase complex, targeting Nrf2 for proteasomal degradation and maintaining low nuclear Nrf2 levels. Reactive oxygen species and electrophilic compounds—whether generated endogenously during metabolic stress or derived from environmental stressors—modify critical cysteine residues on KEAP1, disrupting its interaction with Nrf2. Stabilized Nrf2 then translocates to the nucleus, where it heterodimerizes with small Maf proteins and binds antioxidant response elements (AREs) in the promoters of its target genes, driving transcription of enzymatic antioxidants (SOD, CAT, GPx, GR, GST), glutathione biosynthesis enzymes (GCLC, GCLM, GSS), and NADPH-regenerating enzymes (G6PD, ME1). This pathway is especially relevant in the multistress context because thermal, hypoxic, osmotic, and radiation-induced stressors all converge on KEAP1 cysteine sensors.

The GSSG/tGSH ratio (ratio of oxidized to total glutathione) is a direct and sensitive indicator of glutathione redox balance. Its increase reflects the conversion of GSH to GSSG under oxidative pressure and is informative as a metric of redox status, regardless of whether concurrent enzyme activity measurements are available. That said, the mechanistic picture is more complete when enzyme data are included. Glutathione reductase (GR) activity, as well as the activity of NADPH-generating enzymes, quantifies the regenerative capacity of the system. Glutathione S-transferase (GST) activity, which conjugates electrophilic compounds and lipid peroxidation products to glutathione, provides complementary information on the detoxification flux through the glutathione system. GST is consistently among the responsive antioxidant endpoints across the studies reviewed here [122,128,211], particularly under stressor combinations involving heavy metals, pollutants, or acidification, and its measurement alongside the GSSG/tGSH ratio substantially strengthens the interpretation of glutathione data in multistress contexts.

An additional layer of regulation increasingly recognized in the stress response involves epigenetic mechanisms. Chromatin remodeling, CpG methylation of antioxidant gene promoters, and stress-responsive non-coding RNAs can modulate the transcriptional accessibility of Nrf2 target genes and other redox regulators, shaping how a cell responds to stress signals. Notably, such epigenetic modifications can be induced by environmental stressors and may persist beyond the initial exposure, potentially contributing to acquired stress tolerance and cross-protective effects observed across stressor combinations. A full treatment of these mechanisms is beyond the scope of this review, but they constitute an emerging frontier relevant to understanding the molecular memory of stress in ecophysiological contexts.

### 5.2. Antioxidants and Multiple Stress

Antioxidant systems are part of the core cellular stress response to stressful environments and have been investigated in the response to diverse abiotic and biotic stressors. Abiotic stressors include fluctuations in temperature, oxygen availability, salinity, acidity, radiation exposure, and pollutants (see Section 2). Biotic stressors include exposure to microorganisms and social stress. Understanding how organisms respond to environmental stressors is a central goal in ecophysiology and comparative biochemistry, yet only recently have multistressor approaches begun to receive broader attention within the scientific community.

Studying multiple stressors simultaneously is especially relevant to redox biology, given the central role of oxidative stress as a convergent mechanism underlying response to diverse environmental challenges. For example, both thermal stress and hypoxia can disrupt mitochondrial function and lead to ROS overproduction [212,213]. Some environmental stressors can induce secondary, “hidden” stressors at the cellular level, resulting in “occult” interactions that are not immediately apparent. For example, in many aquatic ectotherms, exposure to thermal stress increases metabolic rates and oxygen demand while simultaneously reducing oxygen solubility in water and impairing oxygen transport. In zebrafish, for example, this mismatch can lead to insufficient oxygen availability, particularly in metabolically active organs like the brain [214]. Thus, what appears to be a singular thermal stressor can in fact functionally trigger a response to hypoxia, which may activate distinct cellular pathways, including redox-sensitive transcription factors, and shift the redox balance toward oxidative distress [215]. The triggering of such cellular stress responses, induced by temperature but mechanistically linked to oxygen limitation, further complicates the interpretation of animal responses to environmental stressors.

Mitochondria occupy a central position in the cellular integration of multistressor inputs. As the primary site of oxidative phosphorylation, mitochondria are simultaneously the main source of endogenous ROS and a primary target of environmental stressors. Both temperature elevation and hypoxia impair electron transport chain function. Beyond their role in ROS generation, mitochondria are also the gatekeepers of the intrinsic apoptotic pathway. When mitochondrial membrane potential collapses under sustained oxidative or energetic stress, programmed cell death is initiated. This multifaceted role as metabolic hub, ROS amplifier and apoptosis initiator makes mitochondrial integrity a key determinant of whether an organism can trigger a stress response or instead progresses to cell death. However, the full treatment of mitochondrial signaling under multistress conditions extends beyond the primary scope of this review.

It is worth noting that, while different stressors engage somewhat distinct primary mechanisms of ROS generation (electron leak from the respiratory chain is enhanced during hypoxia and reoxygenation, heavy metals promote hydroxyl radical formation through Fenton and Haber–Weiss chemistry, and UV-B drives direct photo-oxidation and singlet oxygen production), these associations should not be taken to imply that each stressor produces an exclusive “radical signature” in vivo. In living organisms, multiple ROS-generating systems operate simultaneously, and their relative contributions shift dynamically with stressor intensity, tissue type, and metabolic state. Under combined stress conditions in particular, the intracellular ROS landscape reflects overlapping inputs that are difficult to deconvolute.

### 5.3. Cases on the Effects of Multiple Stressors on the Redox Metabolism (Laboratory Studies)

We examine herein selected examples that examine the impact of environmental stressors on various organisms, particularly focusing on the effect of combined stressors on oxidative stress responses (Table 5). Many studies have investigated how xenobiotics, elevated temperatures, acidification, and hypoxia, individually and in combination, affect animals, often measuring antioxidant enzyme activities (or transcript levels) and oxidative stress markers as indicators of redox balance and activation of cellular stress responses. These studies highlight the occurrence of additive and non-additive interactions (synergistic or antagonistic effects) in complex environmental scenarios compared to single-stressor exposures. Moreover, they also indicate that biochemical responses depend on the specific stressor, its intensity/concentration, duration of exposure, species and tissue.

The examples presented next are all from studies of laboratory simulations of multiple stressors. In the study by De Zoysa and coworkers [126], a transcriptional analysis of selected endogenous antioxidant genes was conducted in the gills of disk abalones (*Haliotis discus discus*) exposed to three isolated abiotic stressors: elevated temperature (28 °C), reduced salinity (25%), and hypoxia. Notably, selenium-dependent glutathione peroxidase (SeGPX) showed a remarkable > 10-fold induction across all stress conditions. However, the analysis of other transcripts revealed that abalones mount a stressor-specific antioxidant response to these environmental challenges. In thermally stressed animals, CuZnSOD and TRX-2 were downregulated. Low salinity elicited a broader upregulation of antioxidants than the other stressors, with all assessed transcripts (MnSOD, CuZnSOD, CAT, TPx, SeGPx, and TRx-2) being upregulated. Hypoxia, on the other hand, selectively induced the expression of MnSOD, CAT, TPx, and SeGPx. These findings from isolated stressors underscore that, although endogenous antioxidants participate in the core cellular stress response [141] and their expression is often regulated by common antioxidant response elements [216], there is a stressor-specific mobilization of specific antioxidants. This fine-tuned and context-dependent mobilization of endogenous antioxidants is further complicated when stressors are combined.

Matoo and colleagues [217] investigated how two estuarine bivalve species, the eastern oyster (*Crassostrea virginica*) and the hard-shell clam (*Mercenaria mercenaria*), respond to elevated temperature (+5 °C) and CO_2_ levels (~800 ppm) alone or in combination, with a particular focus on oxidative damage markers. Through short- (2 weeks) and long-term (up to 15 weeks) exposures, the authors evaluated oxidative damage markers (protein carbonyls, MDA, and 4-HNE protein conjugates) in muscle. These redox biomarkers showed complex and species-specific responses. Clams showed decreased antioxidant capacity following long-term hypercapnia and no signs of oxidative damage. Oysters, on the other hand, displayed a transient but marked accumulation of oxidative damage after 2 weeks of hypercapnia at control temperature, with increases in protein carbonyls and lipid-derived adducts. Interestingly, this oxidative response was attenuated when elevated temperature was combined with high CO_2_, suggesting antagonistic effects of the two stressors. In clams, warming alone (without hypercapnia) caused lipid peroxidation after 15 weeks, but this effect was mitigated by concurrent exposure to elevated CO_2_. Ultimately, the study concluded that moderate warming and acidification do not lead to persistent oxidative stress in these bivalves. The findings suggest that any deleterious effects observed under these conditions, such as temperature-induced mortality, may be driven by mechanisms other than redox imbalance. Alternatively, the weeks-long readout might have missed earlier adjustments in redox metabolism that might have occurred.

Freitas et al. [211] investigated how temperature and mercury (Hg) interact to affect oxidative stress responses in the marine mussel *Mytilus galloprovincialis*. Mussels were first subjected to different thermal regimes (control 17 °C and warming 21 °C) and then maintained at 21 °C or transferred from 21 °C to 17 °C with exposure or absence of Hg (0.25 mg/L). The study aimed to clarify how environmental conditions, particularly thermal history, modulate physiological and biochemical responses to a common metal pollutant. Overall, Hg exposure increased LPO, consumed GSH, caused glutathione oxidation and induced the activity of several antioxidant enzymes (catalase, SOD, GPX and GST). The magnitude of these effects was modulated by temperature, which affected the bioaccumulation of Hg in mussels. Notably, mussels previously acclimated to 21 °C and then transferred to 17 °C displayed elevated LPO and decreased SOD activity even without Hg when compared to control animals maintained at 17 °C, suggesting that thermal history and temperature shifts can disturb redox balance, even in the absence of contaminants. These findings illustrate that stress responses in marine bivalves are not simply dependent on the simultaneous exposure to stressors, but the response to one stressor can be modulated by prior exposure to other stressors (a phenomenon called cross-tolerance). This is illustrated by a vast body of literature that indicates that exposure to a single stressor can enhance tolerance to subsequent challenges of the same nature, such as increased cold tolerance in animals acclimated to low temperatures [218] or ischemic preconditioning improving resistance to ischemic injury [219,220]. Similarly, cross-protective effects are also observed across distinct stressors, as exemplified by heat shock preconditioning improving outcomes following ischemia/reperfusion [221], and by hypoxia exposure enhancing the cardiovascular capacity of fish to withstand acute thermal stress [222], amongst other examples.

Sui et al. [122] investigated the effects of combined acidification and hypoxia on redox metabolism in the thick-shell mussel *Mytilus coruscus*. Mussels were exposed for 72 h to three pH levels (8.1, 7.7, and 7.3) and two dissolved oxygen concentrations (6.0 and 2.0 mg/L). The study measured a suite of oxidative stress markers and endogenous antioxidants, including the activities of SOD, CAT, and GPX, as well as MDA concentration in both gill and hemolymph samples. The results revealed that hypoxia exerted stronger effects on antioxidant enzyme activities than acidification alone. Notably, significant interactions among pH, DO, and exposure time were detected for SOD and CAT, suggesting dynamic regulation of these enzymes in response to combined stress. Both decreased pH and low DO induced similar oxidative responses in *M. coruscus*, and their combined exposure mostly resulted in additive effects, suggesting an increased energetic demand that could ultimately compromise organismal fitness.

González-Ruiz et al. [127] investigated the redox response of white shrimp (*Litopenaeus vannamei*) subjected to high temperature (35 °C), hypoxia (<2 O_2_ mg/L), and subsequent reoxygenation, with a particular focus on the mitochondrial manganese superoxide dismutase (mMnSOD). Hypoxia under the control temperature (28 °C) did not elicit significant changes in mitochondrial SOD activity or mMnSOD transcript levels in both gills and hepatopancreas. However, the exposure to both heat and hypoxia induced the upregulation of mMnSOD transcripts in both tissues. At the enzyme activity level, mitochondrial SOD activity remained unaffected by the combination of heat and hypoxia in both tissues, whereas heat alone induced its activity in the gills. The findings suggest a synergy between thermal and hypoxic stress, leading to mMnSOD transcriptional activation in the gills and hepatopancreas. Notably, hypoxia antagonized the heat-induced SOD activity specifically in the gill tissue at this timepoint. Moreover, the discrepancies between gene expression and enzyme activity suggest possible post-transcriptional regulation or time-dependent dynamics in antioxidant activation, since these were assessed at single exposure time-points. This illustrates that, although RNA levels, protein abundance and protein function are all valuable non-redundant experimental measurements [223], an experimental design aiming to reveal the molecular mechanisms underlying cellular stress responses should consider the temporal disconnection between protein and transcript levels [223] and include multiple time-points to accommodate it.

In an effort to understand animal stress response in an ecologically realistic scenario, Khan et al. [128] investigated the interactive effects of three abiotic stressors: acidification (pH 7.7), hypoxia (2 mg O_2_/L), and warming (30 °C) on the thick-shell mussel *Mytilus coruscus* over a 30-day period. The authors assessed antioxidant enzymes (SOD, CAT, GPX, GST), glutathione, MDA, and total antioxidant capacity in the gills. The general trend was that exposure to multiple stressors induced an activation of endogenous antioxidants (SOD, CAT, GPX, GST, and GSH), as well an increase in MDA levels, by day 15. However, by day 30, the activities of CAT and GPX declined. The analysis of specific redox variables revealed that the responses were not simply additive, with the interactions between stressors producing complex outcomes and some parameters showing synergistic effects initially that transitioned to antagonistic or mitigated responses over time. This study reinforces the idea that the nature and magnitude of the response to interacting environmental stressors can vary with exposure duration and intensity.

Bomble and Nath [224] explored how levels of ROS and reactive nitrogen species (RNS), oxidative stress markers, and antioxidant enzyme activity are affected by desiccation (animals placed on dry paper in <5% relative humidity ambient), heat (40 °C), and starvation (72 h), both individually and in pairwise combinations, in larvae of *Chironomus ramosus*. The time of exposure to each stress was set to the time that caused 20% mortality. The general trend was that, individually, all stresses induced: (i) reactive species production; (ii) oxidative damage to lipids and proteins; (iii) activation of SOD, catalase and GPX activities; (iv) total antioxidant capacity; (v) and a reduction in GR activity. Moreover, the pairwise combination of them had a general simple additive effect (e.g., the effect of desiccation plus heat was greater than that of either stressor alone for several variables) or no effect (e.g., the effect of desiccation plus starvation was similar to that of desiccation alone). The authors advanced to investigating the ternary combination of these stressors, while also assessing them individually and pairwise, this time using larvae of *Drosophila melanogaster* [225]. Dehydration and starvation followed the same protocol of the previous studies, whereas heat stress was set to 37 °C. Again, the time of exposure to each stress was set to 20% mortality. The general trend, again, was that individually the stressors induced an antioxidant response to increased ROS and RNS production and oxidative damage, with dehydration and dehydration plus hypoxia causing the strongest biochemical changes among isolated and pairwise stressor combinations. For most biochemical parameters, the pairwise stressor combinations exerted a synergistic effect. On the other hand, when animals were exposed to the three stressors, a simple additive effect was observed. Taken together, these two studies illustrate that while individual stressors trigger oxidative damage and upregulate antioxidant defenses, the magnitude and nature of interactions depend on the number and identity of co-occurring stressors. For example, pairwise stressor combinations led to synergistic effects, suggesting that the redox balance was more challenged than predicted from single exposures. In contrast, ternary stressor exposure tended to yield additive responses, possibly reflecting a physiological limit beyond which additional stress no longer amplifies the redox response. Thus, synergism is not guaranteed to follow the number of stressors. Instead, complex compensatory mechanisms or resource limitations may limit antioxidant responses when stressors accumulate.

Kim et al. [226] investigated how simultaneous exposure to acidification and thermal stress affects oxidative stress and apoptosis in the abalone *Haliotis discus hannai*. In a 5-day experiment, animals were exposed to combinations of two pH levels (8.1 and 7.5, representing control and acidification) and three temperatures (15 °C, 20 °C, and 25 °C). The authors assessed hydrogen peroxide and lipid peroxidation levels in the hemolymph and determined transcript levels of SOD and CAT in the hepatopancreas. The results revealed that combined exposure to elevated temperature and low pH had synergistic effects. Animals in dual-stressor treatments showed higher levels of oxidative damage and significantly increased mRNA expression of SOD, CAT, and caspase-3, indicating antioxidant defense activation and apoptosis. These responses were markedly stronger in the combined stressor groups than in groups exposed to temperature or acidification alone, suggesting that the interaction between warming and acidification exacerbates physiological stress. Noteworthily, a similar pattern was observed for both lowering the temperature or increasing it, indicating that not only warming but cold stress has an interactive effect with acidification.

Ding et al. [227] investigated the interactive effects of pH (7.0, 8.0, 9.0) and ammonia (0.02, 1.02, 5.10, 10.20 mg/L) on the marine gastropod *Babylonia areolata*, commonly known as the spotted babylon. Over the course of the experiment, the researchers evaluated the redox metabolism by measuring antioxidant enzyme activities. The results showed that the combined stress of altered pH and elevated ammonia levels triggered a stronger antioxidant response than either factor alone. Specifically, the interaction between pH and ammonia led to increased activities of antioxidant enzymes; GPX, CAT, and SOD activities initially increased but later declined. Such an increase in antioxidant levels followed by a decline or return to baseline levels is also observed in other scenarios of exposure to multistressors, as in the case of the thick-shell mussel [128]. The observed patterns suggest that *B. areolata* initiates a response aimed at maintaining redox homeostasis under multiple environmental stressors, but the magnitude and trajectory of these responses vary by enzyme type and exposure duration.

Together, this set of selected laboratory studies illustrates both the growing diversity of experimental designs in redox ecophysiology and the difficulty of drawing universal conclusions when stressors, species, and endpoints vary so widely. A consistent message across studies is that the antioxidant response to environmental stressors is fundamentally context-dependent: no single antioxidant enzyme or biomarker reliably captures the overall redox state across taxa, stressor types, and exposure regimes, because different components of the antioxidant network are independently regulated by distinct transcription factors, operate within different cellular compartments, and are subject to post-translational regulation that can decouple enzyme activity from transcript levels. Three recurring patterns stand out. First, responses to combined stressors are frequently non-additive, with synergistic, antagonistic, or mitigating interactions occurring more often than simple additive effects. Second, the nature of these interactions is strongly context-dependent, varying with stressor intensity, duration, sequence of exposure, tissue examined, and prior stress history—as illustrated by the temporal shift from antioxidant induction to depletion in *Mytilus coruscus* [128] and *Babylonia areolata* [227], and by cross-stressor protection in mussels [211] and fish [228]. Third, discrepancies between transcriptional, enzymatic, and oxidative damage readouts are common, highlighting temporal uncoupling and regulatory trade-offs within redox networks [223].

**Table 5:** Summary of selected laboratory studies examining antioxidant and redox responses to combined environmental stressors in aquatic and semi-aquatic animals. Experimental design indicates the stressor combination approach. Isolated: single stressor tested independently; pairwise: two stressors applied simultaneously; pairwise, sequential: two stressors applied in sequence; ternary: three stressors combined; isolated + pairwise or isolated + pairwise + ternary: multiple combination levels tested within the same study. Interaction type refers to the direction of the combined stressor effect relative to individual stressor responses. Fitness/survival outcomes are absent from most studies.

Species	Stressors	Stressor Levels	Design	Exposure Duration	Tissue	Endpoints Measured	Key Redox Findings	Interaction Type	Reference
*Crassostrea virginica* (eastern oyster)	Temperature, CO_2_	27 °C (ctrl 22 °C) 800 ppm CO_2_ (ctrl 400 ppm)	pairwise	2, 8, and 15 wks	Muscle	TAC, protein carbonyls, MDA, 4-HNE	TAC increased after 15 wks only in warming + CO_2_. Transient elevation of protein carbonyls and lipid adducts at 2 wks under hypercapnia alone. Attenuated when warming was combined with CO_2_ (antagonistic). No persistent oxidative stress under long-term combined exposure.	Antagonistic (warming + CO_2_ attenuated hypercapnia-induced oxidative damage)	[217]
*Mercenaria mercenaria* (hard-shell clam)	Temperature, CO_2_	27 °C (ctrl 22 °C) 800 ppm CO_2_ (ctrl 400 ppm)	pairwise	2, 8, and 15 wks	Muscle	TAC, protein carbonyls, MDA, 4-HNE	TAC decreased after 15 wks in hypercapnia alone and in warming + CO_2_. Warming alone elevated MDA at 15 wks; mitigated by concurrent CO_2_. Minimal persistent oxidative stress under combined conditions.	Antagonistic (CO_2_ mitigated warming-induced LPO)	[217]
*Mytilus coruscus* (hard mussel)	pH, hypoxia	7.7, 7.3 (ctrl pH 8.1) 2.0 mg/L (ctrl 6.0 mg/L)	pairwise	12, 24, 48, and 72 h	Gills, hemolymph	SOD, CAT, GPX, MDA	Hypoxia had greater effects than acidification alone; combined response additive for most endpoints; significant pH × DO × time interactions for SOD and CAT; similar antioxidant patterns in gills and hemolymph.	Additive	[122]
*Mytilus coruscus* (hard mussel)	Temperature, pH, hypoxia	30 °C (ctrl 20 °C) 7.7 (ctrl 8.1) 2 mg/L (ctrl 6 mg/L)	ternary	15 and 30 days	Gills	SOD, CAT, GPX, GST, GSH, MDA, TAC	Antioxidants (SOD, CAT, GPX, GST, GSH) and MDA initially increased at day 15 then declined at day 30; TAC dropped at day 30; GSH increased with all three stressors throughout.	Complex non-additive interactions	[128]
*Mytilus galloprovincialis* (Mediterranean mussel)	Temperature, mercury	21 °C (17 °C ctrl) Hg 0.25 mg/L	pairwise, sequential	14 days temperature, 28 days Hg exposure	Gills	LPO, GSH, GSSG, CAT, SOD, GPX, GST	Hg induced oxidative stress at both temperatures: elevated LPO, GSSG, SOD, CAT, GPx, GST; depleted GSH. The 17 °C mussels accumulated more Hg and showed greater oxidative damage (higher LPO, SOD, GPx, GST) than 21 °C; CAT and GSH depletion did not differ between temperatures.	Cross-tolerance	[211]
*Mytilus galloprovincialis* (Mediterranean mussel)	Hyposalinity, temperature	20, 28 ppt (ctrl 34 ppt) 20, 25 °C (ctrl 17 °C)	pairwise, sequential	7 days hyposalinity, 2.5 h temperature	Gills	SOD	SOD activity influenced by temperature, salinity, and their interaction. At 17 °C (ctrl): no salinity effect on SOD. At 20 °C (moderate heat): highest SOD activity overall—>4-fold increase at 20 °C + 34 ppt and >3-fold at 20 °C + 20 ppt vs. control; 20 °C + 28 ppt was similar to 17 °C and 25 °C groups. At 25 °C (extreme heat): SOD returned to control levels regardless of salinity—extreme heat did not sustain the antioxidant induction seen at moderate heat.	Non-monotonic: moderate temperature (20 °C) maximally induces SOD; extreme temperature (25 °C) suppresses it back to control levels regardless of salinity; no salinity effect on SOD at control temperature	[229]
*Haliotis discus discus* (disk abalone)	Temperature, salinity, hypoxia	28 °C (ctrl 20 °C), 25% (ctrl 32%), hypoxia	isolated, not combined	1, 2, 4, 8, 12, and 24 h	Gills	MnSOD, CuZnSOD, CAT, TPx, SeGPx, TRx-2	SeGPx highly induced by all stressors; CuZnSOD and TRx-2 down in thermal; all transcripts up under low salinity; hypoxia selectively induced MnSOD, TPx, SeGPx	None, single-stressor design	[126]
*Haliotis discus hannai* (Pacific abalone)	Temperature, pH	15°, 25 °C (ctrl 20 °C), 7.5 (ctrl 8.1)	pairwise	1, 3, and 5 days	Hemolymph, hepatopancreas	H_2_O_2_, LPO, SOD, CAT	All stressors (individually and combined) elevated H_2_O_2_ and MDA in hemolymph and upregulated SOD, CAT, and caspase-3 transcripts in hepatopancreas relative to control. Temperature acted faster than acidification: HT (25 °C) elevated H_2_O_2_ and SOD/CAT mRNA from day 1; for OA alone (pH 7.5, 20 °C), effects were delayed to day 3–5.	Additive, Temperature-dominated	[226]
*Babylonia areolata* (spotted babylon)	pH, ammonia	7.0, 9.0 (ctrl 8.0) 1.02, 5.10, and 10.20 mg/L (ctrl 0.02 mg/L)	pairwise	24, 48, 72, and 96 h	Soft tissues	SOD, CAT, GPX, POD	Stressors induced a biphasic antioxidant response: enzyme activities (SOD, POD, CAT, GPx) increased through 72 h then declined by 96 h, suggesting initial compensatory induction followed by capacity depletion. SOD increased with ammonia at pH 8.0 and 9.0; most pronounced at pH 8.0–9.0 + 10.20 mg/L; NO significant change at pH 7.0. POD increased gradually through 96 h; higher at pH 9.0 than 8.0. CAT and GPx increase then decrease; peak at 72 h; greater changes at pH 9.0.	pH-direction-dependent: alkaline pH amplifies ammonia toxicity (NH_3_ speciation); antioxidant response biphasic—induction through 72 h, depletion by 96 h	[227]
*Litopenaeus vannamei* (Pacific white shrimp)	Temperature, hypoxia, reoxygenation	35 °C (ctrl 28 °C), <2 mg/L (ctrl 5.3 mg/L), reoxygenation	pairwise	24 h hypoxia, 24 h hypoxia + 1 h reox.	Gills, hepatopancreas	mMnSOD, SOD	Heat + hypoxia synergistically induced mMnSOD transcript in gills and hepatopancreas; hypoxia antagonized heat-induced SOD activity in gills; discrepancy between transcript and enzyme activity suggests post-transcriptional regulation	Synergistic (transcriptional level); antagonistic (enzyme activity in gills)	[127]
*Litopenaeus vannamei* (Pacific white shrimp)	Temperature, hypoxia, reoxygenation	35 °C (ctrl 28 °C), 1.53 mg/L (5.28 mg O_2_/L ctrl), reoxygenation	pairwise	24 h hypoxia, 24 h hypoxia + 1 h reox.	Gills, hepatopancreas	GPx, CAT, cMnSOD, GSH, GSSG, GSH/GSSG ratio, H_2_O_2_	cMnSOD (transcript) synergistically induced by temperature × hypoxia in both gills and hepatopancreas. CAT (transcript): reoxygenation drives expression in gills; temperature drives expression in hepatopancreas during hypoxia. CAT activity in gills: no overall effect. Hepatopancreas CAT activity strongly induced by temperature. GPx (transcript): no significant change in gills. GPx activity: unaffected in gills; increased by reoxygenation and temperature in hepatopancreas. SOD activity (hepatopancreas): increased by temperature in normoxia; hypoxia antagonized this effect. Glutathione: GSSG changed only with temperature, not oxygen condition. Gills: GSSG decreased at 35 °C (GSH/GSSG ratio up). Hepatopancreas: GSSG increased at 35 °C (GSH/GSSG ratio unchanged).	Strongly tissue- and endpoint-specific: cMnSOD synergistic (temperature × hypoxia); SOD activity antagonistic in hepatopancreas (hypoxia blunts temperature effect); CAT activity antagonistic in gills (H-35 < H-28); glutathione redox state driven by temperature alone, oxygen-independent	[129]
*Larimichthys crocea* (large yellow croaker)	Hypoxia, copper	3.0 mg/L (pre-exposure), 168 µg/L Cu (challenge)	pairwise, sequential	48 h hypoxia + 48 h copper in normoxia	Liver	ROS, MDA, SOD, CAT, GPx, GSH, TAC, HIF-1α, Nrf2, FoxO3	Cu alone induced oxidative damage; hypoxia pre-exposure upregulated hepatic antioxidant defenses (via Nrf2), reducing Cu-induced ROS and MDA; reduced Cu content in pre-exposed fish	Cross-tolerance (not simultaneous combined exposure)	[228]
*Chironomus ramosus* (non-biting midge)	Desiccation, temperature, starvation	<5% RH 40 °C 72 h starvation	isolated + pairwise	Time to 20% mortality (stressor-specific)	Whole larvae	ROS, RNS, LPO, protein carbonyls, SOD, CAT, GPX, GR, TAC	All individual stressors induced ROS, oxidative damage, and antioxidant enzyme induction; GR activity reduced across stressors; pairwise combinations mostly additive; desiccation + heat was the most potent combination	Additive (pairwise combinations)	[224]
*Drosophila melanogaster* (fruit fly)	Desiccation, temperature, starvation	<5% RH, 37 °C starvation	isolated + pairwise + ternary	Time to 20% mortality (stressor-specific)	Whole adults	ROS, RNS, LPO, protein carbonyls, SOD, CAT, GPX, GR, TAC	Individual stressors induced oxidative damage; pairwise combinations synergistic for most parameters; dehydration + heat most potent pairwise; ternary combination was additive—suggesting physiological ceiling beyond which additional stressors do not amplify the response	Synergistic (pairwise); additive (ternary)	[225]

Abbreviations: 4-HNE, 4-hydroxynonenal; CAT, catalase; cMnSOD, cytosolic manganese superoxide dismutase; CuZnSOD, copper–zinc superoxide dismutase; FoxO3, forkhead box O3 transcription factor; GPx, glutathione peroxidase; GR, glutathione reductase; GSH, reduced glutathione; GSH/GSSG, glutathione redox ratio; GSSG, glutathione disulfide (oxidized glutathione); H_2_O_2_, hydrogen peroxide; HIF-1α, hypoxia-inducible factor 1-alpha; LPO, lipid peroxidation; MDA, malondialdehyde; mMnSOD, mitochondrial manganese superoxide dismutase; MnSOD, manganese superoxide dismutase; Nrf2, nuclear factor erythroid 2-related factor 2; POD, peroxidase; protein carbonyls, protein carbonylation products; RNS, reactive nitrogen species; ROS, reactive oxygen species; SeGPx, selenium-containing glutathione peroxidase; SOD, superoxide dismutase; TAC, total antioxidant capacity; TPx, thioredoxin peroxidase; TRx-2, thioredoxin-2.

These findings also expose the fact that it is not yet possible to answer definitively which stressor combinations consistently produce synergistic, additive, or antagonistic oxidative responses across taxa. The studies reviewed here differ substantially in species, tissue, stressor identity, intensity, duration, and endpoints, making cross-study comparison of interaction magnitudes unreliable without a formal meta-analytic framework with standardized effect sizes. What the available evidence does suggest is that temperature × hypoxia is the combination with the most consistent evidence for synergism at the redox level, with independent studies in crustaceans and bivalves reporting stronger-than-additive induction of antioxidant enzymes and oxidative damage [127,128,129,226]. These studies are, by necessity, attempts to reconstruct natural events inside the lab; they cannot fully reproduce the environmental and ecological complexity of real events, but they provide essential information about the biochemical processes expected to occur in nature. A critical and largely unaddressed gap across this body of work is the lack of fitness-level endpoints alongside biochemical markers. The majority of reviewed studies measure antioxidant enzymes and oxidative damage markers without concurrent assessment of mortality, scope for growth, or reproductive success. Vasquez et al. [229] represent one exception, reporting metabolic rate and clearance rate alongside antioxidant enzyme activities in *Mytilus galloprovincialis* under combined stressors.

## 6. Multistressors’ Effect on Antioxidants: Field Studies

In natural environments, animals are simultaneously exposed to a variety of stressors, whose intensities are governed by temporal cyclicity through daily patterns (such as tidal cycles and light–dark cycles) [84], seasonal [23] and even interannual fluctuations (climatic oscillations such as ENSO/La Niña) [230,231] which challenge the physiological stability of animals [232]. The combined action of multiple stressors under different environmental scenarios may rapidly drive organisms beyond their upper and lower physiological tolerance limits, leading to an excessive formation of reactive oxygen species (ROS) [233]. Consequently, this process can induce structural and functional alterations in key macromolecules, compromise cellular homeostasis, and force organisms to reorganize their energy budget so that resources allocated to growth, reproduction, or storage are diverted towards cellular defense and repair mechanisms [234]. However, the magnitude of these effects depends on the specific combination of stressors, their intensity and duration, as well as the history of exposure and the degree of adaptive plasticity of each species. To counteract this, organisms must deploy rapid and efficient mechanisms—the endogenous antioxidant system—capable of intercepting and controlling ROS at the cellular level to minimize their deleterious effects [204]. Numerous studies have assessed the redox metabolism in organisms collected directly from natural environments and immediately dissected and frozen. In this section, we discuss works on the effects of multistress on endogenous antioxidants in sponges, cnidarians (anemones, corals, sea pens), mollusks (gastropods and bivalves), annelids, arthropods (crustaceans), echinoderms (holothurians, sea stars, and sea urchins) and fish (Table 6). The work of Regoli et al. [235] explored the antioxidant response of the symbiotic sponge *Petrosia ficiformis* along a seasonal gradient on the coast of Paraggi (Portofino, Italy). For this purpose, specimens of this sponge were collected monthly over a one-year period (March 2000 to February 2001) at a depth of 5 m. Data on seawater temperature and solar radiation levels were recorded, and analyses of the antioxidant activity of SOD, CAT, GST, GPx, and GR were performed for both environmentally exposed and non-exposed tissues. According to the authors, “results indicate seasonal changes in antioxidants, with a different pattern in different parts of the sponge: more marked variations in the red outer layer than in the white inner portion.” Catalase was increased in summer in the outer layer, indicating a role of both temperature and solar radiation in the outcomes. Liñán-Cabello et al. [13] conducted quantifications in the coral *Pocillopora capitata* at Boquita Reef (Colima, Mexico), where they evaluated the combined impact of depth and seasonal changes on radiation levels (200/320 W m^−2^) and temperature (27 ± 1.1 °C) on the antioxidant response of this sessile cnidarian. Their results indicate that seasonality significantly influenced the antioxidant mechanisms of this species. During winter, SOD activity was elevated, showing a 57% reduction in summer, whereas the enzymes CAT, GPx, and GR significantly increased their activity during the summer season when environmental radiation increased significantly. As in the above-described study of the sponge *P. ficiformis*, temperature and solar radiation were associated with the redox seasonal changes in the coral.

Similarly, estimates of enzymatic activity levels along a temporal gradient were conducted by Cubillos [69] in the gastropod *Diloma aethiops* and the anemone *Actinia tenebrosa* within an intertidal community located in a mid-latitude zone (45°47′52.4″ S) on the coast of New Zealand over a 27-month period. During this time, quantifications were performed to assess how exposure to environmental radiation (UVB + UVA + PAR: >280–700 nm) and aerial temperature during low-tide periods influenced the antioxidant responses of antioxidant enzymes (SOD, CAT, GPx, GR) and glutathione (GSH, GSSG). The results indicate that seasonality significantly affects animal responses, with the highest antioxidant activities correlating mainly with increases in UV-B radiation and temperature during the summer season. This generated differences not only in oxidative damage levels but also in the antioxidant mechanisms between the two species. The transition from winter to summer resulted in *A. tenebrosa* exhibiting a significant increase in lipid peroxidation, which was accompanied by increases in SOD, CAT, GPx, and GR activities. In contrast, in the gastropod *D. aethiops*, changes in surrounding environmental conditions led to a significant increase in lipid peroxidation, with corresponding increases in antioxidant enzymes. The comparatively smaller increases in antioxidant levels observed in the snail during seasonal transitions highlight its lower tolerance to changes in surrounding environmental conditions, unlike the sessile condition of the anemone. In this study it is again clear that two abiotic factors may act together in the induction of redox responses.

In highly dynamic environments such as estuaries, changes in salinity, temperature, and environmental UV radiation driven by tidal cycles and seasonality have been shown to directly influence not only oxidative damage levels but also the total antioxidant capacity of the symbiotic anemone *Anthopleura hermaphroditica* [23]. During summer, *A. hermaphroditica* exhibits five times more macromolecular damage compared to winter, because of elevated temperature and UV-B radiation levels occurring in the estuary, particularly during daily peaks (13:00–14:00 h). Despite this, the total antioxidant capacity in animals acclimated to winter conditions is 66% higher than in summer animals. In this regard, it is important to note that during winter, estuarine salinity may decrease to values < 22 psu (normal psu is 30 to 32) exposing animals to high levels of stress. Nevertheless, persistence in the estuary is possible because phenolic compound levels increase (67%) during winter, thereby enhancing the defensive capacity of this anemone. In this study, we see three different abiotic stresses influencing the outcome of results.

In a field study conducted by Madeira et al. [236] using the intertidal anthozoan *Veretillum cynomorium* (Troia beach, Portugal, latitude 38°29′), the impact of seasonality (changes in temperature and salinity) on the cellular response during emersion periods (13:00–13:30 h) was evaluated in two tissue sections (rachis/peduncle). Their results indicate that both seasonality and tissue type significantly altered lipid peroxidation levels, with higher damage observed in spring compared to summer (salinity was unchanged between seasons). A similar trend was observed in antioxidant levels, where SOD and CAT significantly decreased during periods of higher temperature (in summer), possibly due to limited energy availability due to investment in reproduction. The lack of determination of solar UV radiation precluded the use of this abiotic factor for the understanding of the biochemical differences in the two seasons.

The following study investigated the effects of seasonal variation associated with the drought period in the Montividiu River (central Brazil) [67]. The fish *Astyanax elachylepis* was evaluated during dry and rainy seasons in both intermittent and perennial stream sites. During drought, reduced water flow confines fish to shallow pools where water quality deteriorates, leading to decreased pO_2_ and increased temperature and conductivity. The authors found that oxidative stress responses were strongly influenced by seasonality, being intensified in intermittent streams. Lipid peroxidation increased significantly during drought in the gills of fish from intermittent sites. In the liver, lipid peroxidation increased during the dry season in both stream types, suggesting a generalized seasonal effect. Antioxidant enzymes also showed seasonal modulation in gills, with various enzymes (CAT, G6PDH, GPx and GR) displaying increased activity in the dry season, with special attention to GPx in intermittent streams during drought. Overall, the results demonstrate that drought-induced confinement and hypoxia increase oxidative stress, which may compromise fish physiological performance and persistence in intermittent tropical streams. In most studies in nature it is very difficult to isolate a particular abiotic factor as an inducer of redox adaptations. However, for this Brazilian fish study, hypoxia in drying streams is a key player for those adaptations.

The multistress effect of surrounding environmental conditions in natural habitats on the antioxidant response has also been evaluated in bivalve mollusks through numerous direct quantifications in different aquatic environments, as well as under cross-transplant conditions. Specifically, Moreira et al. [59] determined oxidative response and antioxidant strategies under multistress conditions (air temperature, water temperature, solar radiation, and oxygen availability were monitored) in the mussel *Brachidontes solisianus* across an in situ tidal cycle (emersion, immersion, emersion) on the southern coast of Brazil. Tidal changes did not induce significant variations in the mussel’s CAT, GST, GR, or G6PDH levels, but they did reveal a significant 60% increase in GSH at the end of emersion, as well as an increase in oxidative damage markers (lipid peroxidation and carbonyl proteins) after 4 h of aerial exposure, reflecting signs of the preparation for oxidative stress (POS) mechanism. The increased levels of GSH in low tide enable mussels to withstand the stress of re-immersion—a reoxygenation-like event. Interestingly, the activation of GSH synthesis in *B. solisianus* happened on a day with high solar UV radiation levels. On a cloudy day (the very next day, with three times less total solar UV levels), there was no increase in GSH or oxidative stress [58]. This result indicates that the redox response was activated by both environmental UV radiation and air exposure (causing functional hypoxia). Interestingly, there were no relevant differences in temperature during the tide cycle on both days, indicating this abiotic factor was not relevant for the recorded biochemical differences. Therefore, air exposure plus UV radiation were the main inducers of mussel redox changes in this case.

Similar observations were carried out in the intertidal mussel *Perumytilus purpuratus* at Calfuco Beach (Valdivia, Chile), where samples were collected from the natural environment at the upper (UL) and lower (LL) limits of its distribution belt every 2 h during a natural tidal cycle [68]. In addition, a reciprocal transplant experiment was conducted between organisms acclimated to UL and LL conditions, which were sampled at the same time intervals as described above. In parallel, levels of UV-B radiation, relative humidity, air temperature, rock temperature, and shell temperature were recorded. The results indicate that upper versus lower intertidal positions differ in aerial exposure, feeding time, and metabolic demand, which helps explain the observed redox patterns; the reciprocal transplant results indicate that *P. purpuratus* can rapidly adjust its antioxidant defenses when moved to a more stressful (upper) microhabitat. This caused upregulation of antioxidants in preparation/response to reoxygenation/emersion, consistent with the POS strategy. The rapid inducibility of antioxidants in transplants indicates high phenotypic plasticity in *P. purpuratus*, allowing metabolic adjustment to abrupt microhabitat changes and contributing to the species’ bathymetric distribution. Moreover, the results also indicate interaction of solar radiation with air exposure for the mussel redox response; temperature was excluded as the primary factor explaining the differences in oxidative response between the two groups because the temperature of the mussel tissues and shells did not show significant differences between the two bathymetric levels (upper and lower) during the study.

As mentioned in Section 3, Pereira et al. [66] conducted a field study at Manguinhos Beach, Espírito Santo, Brazil, to evaluate the multistress effects of temperature and solar radiation (during immersion, emersion, and re-immersion phases of the tidal cycle) on the redox response (lipid peroxidation and activities of SOD, CAT, and GST) of the adult sea urchin *Echinometra lucunter*. The results of this study indicate that periods of aerial exposure, characterized by functional hypoxia, combined with elevated temperature and solar radiation, induced a four-fold increase in GST in the intestine of *E. lucunter*. Lipid peroxidation was elevated in gonads in air exposure, with no change in antioxidants. Temperature increased from 23.5 °C at 5 AM in underwater urchins to 27.8 °C in air exposure at 7 AM (considering air temperature). The 4.3 °C difference was considered to play a role in modulating redox metabolism during aerial exposure, unlike solar radiation, which increased poorly between 5 and 7 AM. These results were also indicative of a POS mechanism in natural conditions for *E. lucunter*.

The multistress effect of solar radiation, ozone levels, and depth was evaluated by Lister et al. [17] using larvae of the tropical sea urchin *Tripneustes gratilla* as a biological model in Aitutaki, Cook Islands (18.85° S, 159.75° E). For this purpose, larvae were cultured in the natural environment at depths of 1 and 4 m, where they were exposed to different types of environmental radiation (PAR [>400–700 nm], PAR + UVA [>320–700 nm], and PAR + UVA + UVB [>280–700 nm]). This was done by employing radiation filters placed above cages with the animals. Temperature was identical at the two different depths. UV-B radiation exposure under shallow-water conditions (1 m) for 24 h generated high levels of cellular damage in urchins, as evidenced by the appearance of abnormalities during pluteus larval development. This effect was correlated with significant increases in SOD, CAT, GPx, and GR levels, as well as protein carbonyls, compared with larvae exposed only to PAR and PAR + UVA treatments at both 1 and 4 m depth. However, biochemical changes were bigger at 1 m than at 4 m. This indicated that less UV-B penetration at 4 m induced less photo-oxidative damage [78,237] as well as antioxidant enzyme adaptation. In this study, it was clear that other abiotic factors did not play significant roles for the alterations in redox metabolism.

In line with the above study, Lister et al. [18] assessed the role of environmental radiation (PAR, UV-A, and UV-B) and sea-ice cover (which is vital for the successful development of these early life stages) on the photo-oxidative response of embryos of the Antarctic sea urchin *Sterechinus neumayeri* at Cape Armitage, McMurdo Sound, Antarctica (77.06° S, 164.42° E). Early developmental stages were exposed to three radiation treatments—PAR, PAR + UVA, and PAR + UVA + UVB—at depths of 1 and 4 m below the surface (both with and without ice cover) over a period of approximately 90 h. The study was conducted during two distinct periods characterized by either low or high levels of atmospheric ozone. The results indicated that embryonic exposure to UV-B radiation (PAR + UVA + UVB treatment) in an environment without surface ice cover generated elevated levels of photo-oxidative damage and significant structural abnormalities, as well as higher SOD and CAT activities compared with embryos in an ice-protected environment. In other words, in open-sea conditions, the embryos’ endogenous antioxidant levels were insufficient to prevent oxidative damage, drawing attention to the relevance of ice cover for UV-B protection. Moreover, the presence of higher or lower levels of ozone in the atmosphere had a modulatory effect on redox metabolism, as it directly affected the UV-B levels reaching the embryos. Temperature, however, had no effect, as it remained constant during both experimental periods. This study also points to a natural limit for antioxidant protection in *S. neumayeri*, which in some instances is insufficient to counteract the effects of elevated environmental radiation.

It is also essential to emphasize that estuaries function as true sinks for heavy metals and other contaminants. Owing to their mixing dynamics, tides, and sedimentation processes, they retain fine particles and organic matter with a high capacity to absorb metals such as cadmium, lead, copper, and mercury [238,239]. This accumulation not only increases their concentration in sediments but also potentially releases them back into the biological system, generating considerable ecological risks [240], and thus making estuaries critical areas for environmental monitoring and management. Anthropogenic inputs through heavy metal deposits and compounds such as pesticides have been extensively studied in various marine invertebrates, where antioxidant responses clearly reflect a wide range of metallic and non-metallic elements, in addition to the interaction of temporal and spatial effects [241,242]. For example, Rodrigues et al. [242] examined the endogenous antioxidant response of the decapod *Carcinus maenas* under different conditions of environmental pollution in the Minho and Lima estuaries along the Iberian coast, across both temporal (interannual) and spatial (upstream and downstream) gradients. Abiotic parameters such as contaminant levels, salinity, conductivity, temperature, pH, and dissolved oxygen in the water column were quantified. Their results indicate that in the polluted estuary (Lima), heavy metal levels in both sediments and soft tissues of the crab *C. maenas* were markedly higher in the summer of 2010 compared with the summer of 2012, suggesting a recovery trend of the estuary during the sampling period. The above is the product of a multifactorial response comprising: (i) a reduction in industrial activity because of an economic restriction that acted as an involuntary remediation measure; (ii) the result of the implementation of environmental protection policies and regulatory frameworks; and (iii) uncontrolled environmental alterations. Although the dry season in 2012 characterized by high temperatures may have generated elevated levels of stress in crabs, the reduction in the “dominant stressor” (pollutants) from 2010 to 2012 may have generated an alleviated condition for the redox machinery.

In addition, the work of Mansour et al. [241] on the *Ruditapes decussatus* clam exhibited the impact of heavy metals (cadmium, copper, iron, lead, and zinc), salinity, temperature, and pH between summer and winter seasons at three sites with anthropogenic impact in Tunis Lagoon (Tunisia). Seasonality had a significant effect on temperature and antioxidant levels: during winter, an increase in redox variables—CAT, GPx, GR, SOD, and lipid peroxidation (60% to 170% increases)—was associated with reproductive status and food availability. On the other hand, very modest redox changes were noticed in summer. Thus, the interaction of trace metals (Cd, Fe, Pb, Zn) in the polluted site of the Southern Lagoon of Tunis (S1, navigation channel) and low temperature observed only during winter periods (10 °C) led to an increase in measurable oxidative damage (MDA), triggering apoptosis, a situation that revealed neurotoxicity due to an inhibition of acetylcholinesterase activity in those animals compared to those unexposed to heavy metals.

**Table 6:** Summary of selected multistress field survey studies examining antioxidant and redox responses to combined environmental stressors in aquatic animals. Experimental design indicates the stressor combination approach. Isolated: single stressor tested independently; pairwise: two stressors applied simultaneously; pairwise, sequential: two stressors applied in sequence; ternary: three stressors combined; isolated + pairwise or isolated + pairwise + ternary: multiple combination levels tested within the same study. Interaction type refers to the direction of the combined stressor effect relative to individual stressor responses. Fitness/survival outcomes are absent from most studies.

Phylum	Species	Stressor	Design	Duration	Tissue	Endpoints Measured	Key Redox Findings	Interaction Type	Author
Porifera	*Petrosia* *ficiformis*	Water T° Salinity Solar radiation	Ternary multiple combination levels	12 months	Symbiocortex (outer tissue) White cortex (inner tissue)	SOD, CAT, GST, GR, GPx TOSC	Symbiocortex has more antioxidant fluctuations than white cortex during summer. CAT and TOSC increase during summer. GPx and Gr levels reduce during summer.	Additive or synergistic interactions between solar radiation and temperature.	[235]
Cnidaria	*Pocillopora* *apitata*	Depth Water T° UV-R	Ternary multiple combination levels	6 months Summer/Winter	Soft tissues of coral fragments	LPO SOD, CAT, GPx, Gr, GST O_2_^−^	Superoxide increases 7.6-fold during summer driven by solar radiation and temperature. Higher TBARS levels in the summer than in the winter.	Additive or synergistic interactions between solar radiation, depth and temperature.	[13]
	*Veretillum cynomorium*	Salinity Air T° Water T° pH Oxygen availability (tidal cycle)	Ternary multiple combination levels	6 h	Rachis Peduncule	LPO SOD, CAT, GST	SOD and CAT levels reduce from spring to summer. MDA levels were significantly lower in the summer. Significant differences in activity between tissues were found for GST and MDA.	Additive or synergistic interactions between solar radiation, temperature, pH, and air/water exposure condition.	[236]
	*Anthopleura hermaphroditica*	Solar radiation Salinity Water T° Oxygen availability (tidal cycle)	Ternary multiple combination levels	Seasonal 12 h tidal cycle	Whole soft tissue	LPO PC TAC	MDA and protein carbonyl levels in summer were 3.72- and 16.51-fold higher than in winter. Mean total antioxidant capacity was 1.7 times higher in winter than in summer. Winter increase was positively correlated with a higher concentration of total phenolic compounds.	Additive or synergistic interactions between solar radiation, temperature, pH, air/water exposure condition.	[23]
	*Actinia* *tenebrosa*	Solar radiation Water T°	Ternary multiple combination levels	Interannual (27 months)	Whole soft tissue	LP PC SOD, CAT, GPx, GR, GSH, GSSG	Oxidative damage follows temporal changes in UV-B levels. Summer-to-winter transition reduces lipid peroxidation and protein carbonyl levels by 60% and 90% respectively. SOD, CAT, and GPx reduced by 75% during winter. Gr activity plays a pivotal role in REDOX balance.	Additive or synergistic interactions between solar radiation, temperature, and aerial exposure condition.	[69]
Mollusca	*Diloma* *aethiops*	Solar radiation Water T°	Ternary multiple combination levels	Interannual (27 months)	Pedal muscle	LPO PC SOD, CAT, GPx, GR, GSH, GSSG	Oxidative damage follows temporal changes in UV-B levels. The transition from summer to winter reduces lipid peroxidation and protein carbonyl levels by 35% and 20% respectively. SOD and CAT get reduced by 50% and 35% during winter.	Additive or synergistic interactions between solar radiation, temperature, and aerial exposure condition.	[69]
	*Brachiodontes solisianus*	Air T° Water T° Solar radiation Oxygen availability (tidal cycle)	Ternary multiple combination levels	Day 1: sunny (14 h.) Day 2: cloudy (14 h.)	Whole soft tissue	LPO PC CAT, GST, GR, GSH	On a sunny day, the 4 h evening air exposure increased TBARS and protein carbonyl levels. On a cloudy day, these damage markers remained at baseline, proving that low-intensity light does not trigger this stress. Sun + air exposure triggered a 1.6-fold increase in GSH, being the hallmark of the POS strategy.	Additive or synergistic interactions between temperature, salinity, UV-R solar radiation and changes in oxygen availability (tidal changes).	[59]
	*Brachiodontes solisianus*	T° Salinity UV-R Oxygen availability (tidal cycle)	Ternary multiple combination levels	13.5 h	Whole soft tissue	LPO PC CAT, GST, GR, G6PD metabolic enzymes: PK, MDH, IDH	TBARS and protein carbonyl increased 7- and 2.3-fold respectively compared to mussels underwater for 7 h. Antioxidant enzymes (catalase, GST, GR, G6PDH) and metabolic enzymes (PK, MDH, IDH) remain stable throughout the tidal cycle.	Additive or synergistic interactions between temperature, salinity, UV-R solar radiation and changes in oxygen availability (tidal changes).	[58]
	*Perumytilus purpuratus*	Air T° Water T° Rock T° Shell T° RH Solar radiation Bathymetry UV-R Oxygen availability (tidal cycle)	Ternary multiple combination levels	12 h	Whole soft tissue	LPO PC TAC	All mussels showed increased lipid damage during aerial exposure. Upper intertidal mussels showed higher levels of lipid peroxidation compared to those in the lower intertidal fringe. Protein carbonyl levels increased by 300% in both groups after 2 h of air exposure. Upper intertidal mussels demonstrated a significantly higher total antioxidant capacity (TAC) at the end of the emersion period (a 33% increase). Lower intertidal mussels moved to the upper intertidal (high stress) show an increase in lipid peroxidation, even higher than native upper intertidal mussels.	Additive or synergistic interactions between temperature, relative humidity, UV-R solar radiation changes in oxygen availability (tidal changes) and bathymetric origin.	[68]
	*Ruditapes decussatus*	Heavy metals Salinity T° pH	Ternary multiple combination levels	Seasonal gradient (summer/winter)	Whole soft tissue	LPO SOD, CAT, GST, GPx, GR	Trace metals (Cd, Fe, Pb, Zn) led to an increase in oxidative damage (MDA). Increased apoptosis was linked to severe oxidative stress that clams could no longer repair. Clams showed a significant upregulation or increase in antioxidant enzyme activities across all polluted sites compared to the reference site. Antioxidant enzyme activities (CAT, SOD, GR) and oxidative damage markers (LPO) were significantly higher in winter than in summer.	Additive or synergistic interactions between heavy metals, salinity, temperature and pH.	[241]
	*Tapes philippinarum* & *Mytilus galloprovincialis*	Water T° Solar radiation	Ternary multiple combination levels	8 months	Digestive gland	CAT, GST, GR GPx, GSH TOSC	CAT, GST, and GPx reached their lowest levels during the summer months in both species. TOSC decreased in mussels from winter to summer. Mussels showed much higher seasonal sensitivity. Their redox status fluctuated more dramatically than the clams.	Additive or synergistic interactions between water temperature and solar radiation.	[243]
Crustacea	*Carcinus* *maenas*	Pollutants Salinity Conductivity Temperature pH Oxygen concentration	Ternary multiple combination levels	24 months	Digestive gland	LPO PC GST, GPx, GR, GSH	Unlike 2012, during 2010 the polluted area of the estuary increased GST and GPx, showing that crabs were in a high-alert defensive state. During 2010, the incapacity of the antioxidant mechanisms triggered elevated levels of lipid peroxidation. Reduction in pollutant levels by 2012 generated a drop in lipid peroxidation and antioxidant enzymes in crabs. GST-bound metal pollutants prevent the formation of radicals that cause oxidative damage.	Additive or synergistic interactions between pollutants, salinity, conductivity, temperature, pH, and oxygen concentration.	[242]
Echinodermata	*Tripneustes gratilla*	Bathymetry Solar radiation	Ternary multiple combination levels	24 h	Entire organism	LPO PC SOD, CAT, GPx, GR	Oxidative damage was significantly higher at depths of 1 m than at 4 m. Exposure to UV-B radiation (at 1 m depth) led to a 2.3-fold increase in developmental abnormalities and a 3.3-fold increase in oxidative damage to proteins. Exposure to UV-B triggered a significant increase in the activity of protective enzymes (SOD, CAT, GPx, Gr).	Additive or synergistic interactions between bathymetric position and solar radiation type (PAR, PAR + UVA, and PAR + UVA + UVB).	[17]
	*Sterechinus neumayeri*	Solar radiation Ozone levels Sea-ice covering	Ternary multiple combination levels	Period 1: 90 h. Period 2: 92 h.	Entire organism	LPO PC SOD, CAT	The ice covering reduces the formation of lipid-hydroperoxides and oxidized proteins by 50% compared to embryos exposed to ultraviolet radiation. SOD and CAT increased in a dose-dependent manner relative to the UV-B exposure. Although these enzymes increased, they were not high enough to prevent abnormalities during early development.	Additive or synergistic interactions between solar radiation type (PAR, PAR + UVA, and PAR + UVA + UVB), ozone levels, ice covering.	[18]
	*Echinometra lucunter*	T° Solar radiation Oxygen availability	Ternary multiple combination levels	Submerged Re-immersion (7 h)	Gonad Intestine	TBARS SOD, CAT, GST	TBARS levels increased by 21% during aerial exposure. TBARS was maintained even after the urchins were re-immersed in water. Unlike the intestine, the gonads did not show any significant increase in antioxidant enzyme activities (GST, CAT, or SOD) during aerial exposure.	Additive or synergistic interactions between temperature, solar radiation, oxygen availability (submerged, emerged and re-immersed condition), and tissue type.	[66]

Abbreviations: CAT, catalase; G6PD, glucose 6-phosphate dehydrogenase; GPx, glutathione peroxidase; GR, glutathione reductase; GSH, reduced glutathione; GSSG, glutathione disulfide (oxidized glutathione); GST, glutathione S-transferase; IDH, isocitrate dehydrogenase; LPO, lipid peroxidation; SOD, superoxide dismutase; PC, protein carbonyls, protein carbonylation products; POS, preparation for oxidative stress; TAC, total antioxidant capacity; TBARS, thiobarbituric acid reactive substances; TOSC, total oxyradical scavenging capacity. T°, temperature; RH, relative humidity; solar radiation, UV-R + PAR (>280–700 nm); UV-R, ultraviolet radiation (>280–400 nm); UV-B, ultraviolet B radiation (>280–320 nm); UV-A, ultraviolet a radiation (>320–400 nm); PAR, photosynthetically active radiation (>400–700 nm).

Taken together, the various studies presented above highlight the value of examining animals directly in their natural habitats. Environmental conditions that represent a multistress scenario for some species may constitute suitable or even optimal conditions for others, emphasizing the strong influence of local environmental context on physiological responses. Coastal zones, estuaries, and rivers are characterized by distinct patterns of environmental variability that impose different selective pressures and drive adaptive responses in resident organisms. Field-based observations make it possible to capture how organisms truly respond to fluctuations in sunlight, temperature, salinity, or pollution—conditions that cannot be fully replicated under laboratory settings.

## 7. Social Stress

In addition to abiotic stressors, the biotic environment can be a major source of stress arising from predator–prey interactions, parent–offspring dynamics, parasite–host interactions, crowding, or competition for mates and other limited resources [244,245,246,247]. In group-living organisms, social interactions frequently generate stress through competition for territories, food, shelter, or dominance rank [248,249]. Importantly, social stress can interact with other stressors such as extreme temperatures, salinity stress or acute hypoxia. Moreover, individuals frequently differ in their physiological state depending on social status or reproductive timing, with associated changes in endocrine profile and metabolic allocation [250,251,252]. Although these life history states are not inherently stressful, they may strongly modulate how organisms respond to environmental stressors by shaping energetic trade-offs and physiological priorities [253,254]. Thus, the social environment, whether acting as a primary source of stress or as a modifier of other stress responses, should be explicitly integrated into the multistressor framework.

### 7.1. Social Interactions as Stressors

Predator–prey interactions, crowding, and competition are examples of biotic stressors that may activate stress responses (e.g., HPA axis, sympathetic nervous system) and lead to increased metabolic rate and ROS production. One of the most common social stressors results from repeated social interactions in group-living animals where individuals compete for social dominance to gain priority access to mates and other resources [255,256,257]. Low social status in many hierarchy-forming animal species, including humans, may lead to compromised physiology and health due to overactivity of the hypothalamic–pituitary–adrenal axis and mitochondrial alterations [258,259]. It appears that the psychosocial stress of low rank resulting from social intimidation, and in some cases aggression, is sufficient for such physiological dysregulation even in the absence of reduced access to resources [260].

While social challenges may alter antioxidant function in a range of peripheral tissues [261,262], attention has been mostly focused on the brain, as this organ is particularly vulnerable to oxidative stress due to its high energetic needs, lipid-rich environment, and relatively modest antioxidant capacity [263]. Consequently, cumulative oxidative damage has been implicated in nervous system disorders (most in biomedical contexts), with a substantial effort directed toward understanding mechanisms that cause and mitigate oxidative damage [264,265]. From a comparative and ecological perspective, however, maintaining redox homeostasis in neural tissue may represent a broadly conserved physiological challenge across group-living taxa experiencing social stress [266,267,268]. Experimental studies in rodents and other species demonstrate that individuals exposed to chronic social defeat exhibit depression-like symptoms, including anhedonia and social withdrawal [269,270,271]. Importantly, these behavioral outcomes are accompanied by increased oxidative stress in discrete brain regions such as the hippocampus, in both mice and rats [272,273]. Glucocorticoids appear to play a central role in this process by promoting ROS generation and impairing antioxidant function in brain regions implicated in mood disorders [269,273,274]. Beyond antioxidant responses, chronic social stress associated with subordination can also influence the expression of heat shock proteins. In mice, social defeat increases expression of heat shock protein 10 (encoded by *hspe1*) in the hypothalamus [275]. Despite their importance in cytoprotective mechanisms, relatively few studies have examined links between social status and heat shock protein expression. In salmonids, social status influenced HSP expression in a time- and tissue-specific manner, with the degree of subordination positively associated with constitutive levels of Hsc70 and Hsc90 in the brain [276].

While most research has focused on the social stress associated with low social status, high social status and social dominance also entail distinct challenges, including the need to constantly defend one’s rank, the risk of social defeat, developing reproductive tissues, and maintaining dominance-related morphological traits [257,277,278]. Dominant individuals are likely to experience intermittent bursts of oxidative challenges linked to defensive behaviors and may therefore require more robust antioxidant responses [279]. These metabolic demands can be exacerbated at higher population densities, where the rates of social conflict over limited resources is increased [253]. Consequently, dominant individuals may activate protective mechanisms to preserve cellular homeostasis. For instance, in the cichlid *Astatotilapia burtoni*, males undergoing social ascent (transitioning to dominant status with larger gonads and bright coloration) exhibit increased endogenous antioxidant defenses [262], consistent with POS-like adaptation for a metabolically more challenging social state [280]. In salmonids, dominant individuals displayed elevated levels of heat shock proteins in the liver compared to control animals that were not allowed to socially interact [276].

Most research on social stress focuses primarily on the effects of social challenges themselves, which is noteworthy because animals in natural environments often experience social stress alongside a variety of abiotic stressors, such as hypoxia, extreme temperature, or altered salinity levels. Some studies, however, have begun to examine how social stress interacts with these additional stressors. For example, in salmonids, both dominant and subordinate individuals failed to mount a HSP response under acute heat stress, in contrast to socially isolated controls, suggesting that social interactions can suppress cellular heat shock responses [281]. In the same study, subordinate fish were less thermally tolerant than dominant individuals, indicating that social status or repeated social stress can compromise resilience to environmental challenges. Future research could test more explicitly how chronic daily social stress shapes an animal’s ability to protect key tissue against acute oxidative insults. For example, it would be informative to examine how individuals with different levels of social subordination respond to repeated intermittent hypoxia, which is a popular paradigm to study cerebral hypoxic-ischemia [117,282,283] while also reflecting ecologically relevant conditions, given the increasing prevalence of hypoxic events in natural habitats.

### 7.2. Life History Context and Multistressor Response

Life history traits, such as age, reproductive stage, personality or coping style, and social status shape physiological responses to oxidative challenges [284,285,286,287,288,289]. As discussed previously, social stress arising from behavioral interactions in dominance hierarchies can shape antioxidant responses, but social status more broadly can influence responses to stressful conditions. This is because social states are associated with distinct physiological profiles that affect protection against oxidative stress. For example, dominant individuals typically exhibit higher testosterone and lower cortisol levels than subordinates, and both hormones play key roles in the regulation of metabolism and antioxidant responses [288]. Similarly, reproductive stage is accompanied by a distinct neuroendocrine profile, which can significantly influence trade-offs between reproduction and antioxidant capacity under both biotic (e.g., crowding) and abiotic stressors (e.g., temperature). For example, breeding females experience increased oxidative challenges during egg maturation but often upregulate antioxidant responses to protect themselves and their offspring [278,280,287,289], a phenomenon called oxidative shielding [290].

Social context can further modulate responses to environmental stressors. Affiliative relationships between partners or between parents and offspring often buffer the negative effects of stress exposure [291,292,293]. Group living may also reduce stress through enhanced food acquisition [294], increased vigilance [295], and division of labor [296]. Supporting this, the Italian riffle dace (*Telestes muticellus*) exhibited lower exercise-induced elevation of muscle oxidative stress when housed in groups, which was associated with upregulated expression of antioxidant genes such as sod1 and sod2 [297]. The role of the social environment as either a source of stress or a modifier of physiological responses to stressors depends on context and the intensity of behavioral interactions. Social subordination, for example, is a normal part of the life history of many species and is often associated with a nonbreeding phase during early life [298,299,300]. In these cases, social status, with its distinct endocrine and metabolic profile, functions primarily as a modifier, shaping how an animal responds to environmental stressors. However, social subordination can become a significant source of social stress, particularly when aggression is excessive or subordination is prolonged, as chronic social stress often has negative physiological consequences [301]. The framework of allostasis and allostatic load is useful for understanding these dynamics: social experience may either increase or decrease allostatic load, thereby influencing how organisms cope with abiotic stressors [302]. A striking example of why the social environment matters in stress adaptation are studies on psychosocial stress and mitochondrial dysfunction [303]. Mitochondria influence social behavior and adaptation to abiotic and biotic stressors [304,305], making mitochondrial function an important mediator between the social environment and how organisms cope with environmental stressors [306]. Many animals are social or live in groups, and even ostensibly solitary species experience brief but consequential social interactions (e.g., mating). Further, biotic factors can influence exposure to abiotic stress by forcing animals in less preferred habitats or alteration of the biotic environment (e.g., crowding may deplete local oxygen levels). Therefore, integrating social context and life history will be an important step in the multistressor framework to study mechanisms of stress resilience and protection against oxidative stress across individuals, populations, and species.

## 8. Perspectives on Multistress Studies and Conclusions

**Figure 4:**
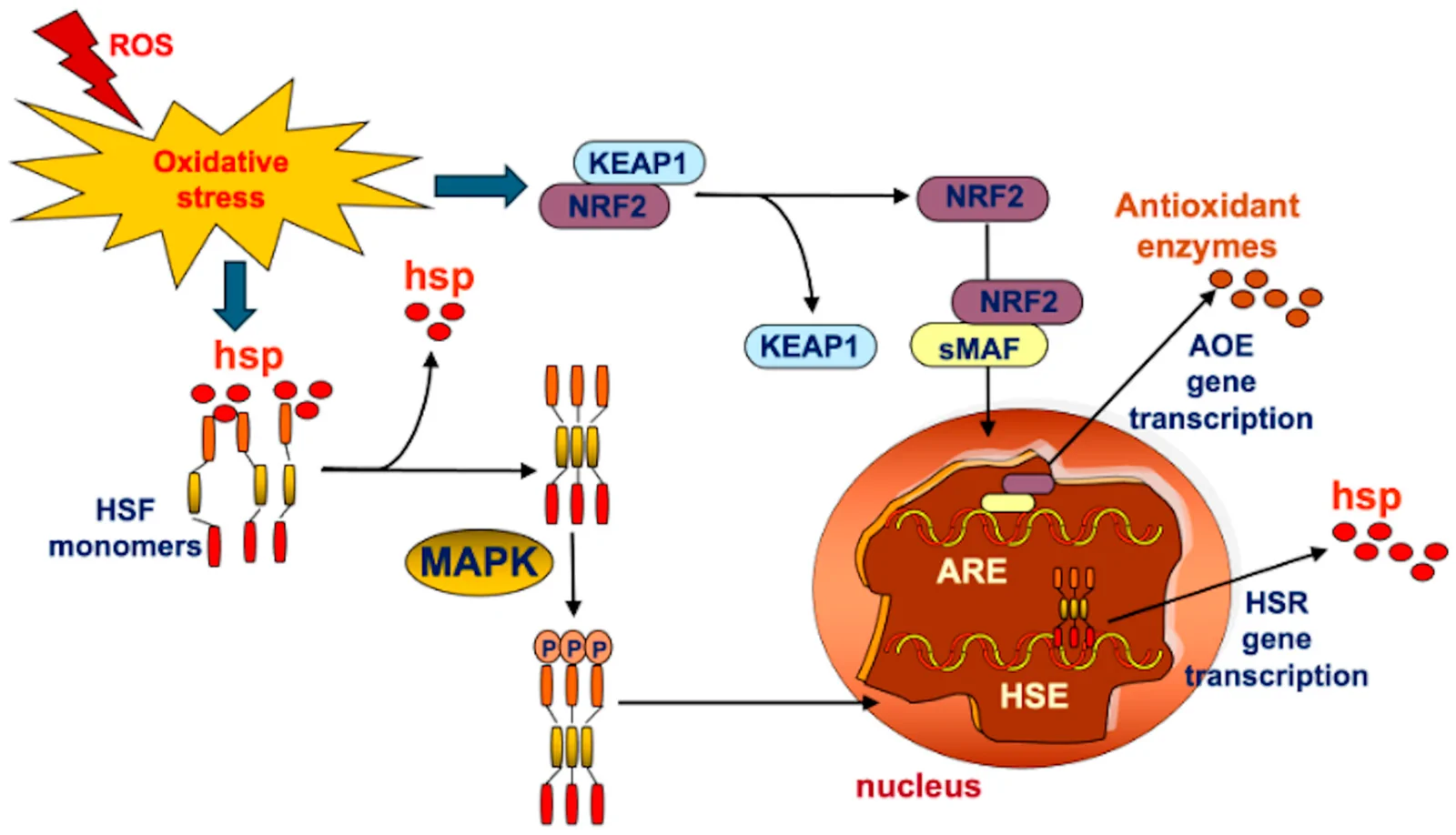
Schematic representation of oxidative stress-induced activation of the heat shock response (HSR) and the KEAP1–NRF2 antioxidant signaling pathway, leading to heat shock protein (Hsps) synthesis and antioxidant enzyme production.

In the context of animal adaptation to environmental stress, we identify four main responses. The first two processes were extensively discussed in the present review: the endogenous antioxidant system and the HSR. Both are induced by an initial increase in ROS formation, followed by activation of MAPKs and transcription factors, promoting the transcriptional activation of antioxidant- and HSR-related genes (Figure 4). Our article has described a number of examples, from various animal groups, where these two mechanisms are activated under stress pressure—biotic or abiotic. More importantly, we present studies in which one or more natural stresses (low oxygen availability, temperature fluctuations, salinity stress, solar UV radiation and social stress), or man-made stresses (pollution), are combined in either laboratory settings or field/natural experiments. The current challenge is to increase the number of studies in nature (analyzing antioxidants and Hsps) so we can investigate how animals respond to stress in the actual natural world.

There is, however, a significant gap in our understanding of animal responses to multiple stresses regarding the two remaining components, especially in the context of comparative biology: the unfolded protein response (UPR) and the DNA repair systems. It is beyond the scope of our work to discuss these responses in full, so we suggest the review by Fulda et al. [147] for an overall view of molecular mechanisms. Two studies in freeze-tolerant wood frogs are of merit to be highlighted: one is related to UPR during freezing, anoxia, and dehydration stresses [307], and the other on DNA repair responses during freezing and anoxia [308]. Even though the stresses were analyzed in isolation, they form a strong foundation for future work using a multistress approach in the study of environmental stress responses in the wood frog. Regarding the UPR specifically, it is especially relevant to multistressor scenarios since temperature, hypoxia, and salinity-induced protein folding impairment activate this pathway [309,310,311]. Crucially, the cytosolic HSR and the UPR are functionally linked through HSPA5/BiP, the canonical ER-resident Hsp70, whose induction bridges both compartmental stress responses [309,310,311]; future multistress studies should therefore monitor cytosolic (Hsp70, Hsp90) and ER-resident (BiP/HSPA5, XBP1s) stress markers in parallel to capture this interface.

Beyond UPR and DNA repair, additional molecular processes stand at the frontier of multistress biology and warrant dedicated investigation. Epigenetic regulation—including chromatin remodeling at ARE and HSE promoter elements, CpG methylation of Nrf2 target genes, and stress-responsive microRNAs—can provide a molecular memory layer that can modulate the transcriptional competence of antioxidant and HSP genes long after the initial stressor exposure [312,313,314,315]. This epigenetic dimension may underlie some of the acquired stress tolerance and cross-protective effects documented in the studies reviewed here, and its investigation is increasingly feasible in non-model organisms. Similarly, the role of mitochondrial dynamics—biogenesis, fission/fusion, and mitophagy [316,317]—as adaptive responses to multistressor-induced energetic and oxidative stress represents a productive area for future research, particularly given the central role of mitochondria as both ROS generators and apoptotic gatekeepers under combined stress conditions.

We encourage future studies examining UPR, DNA repair, epigenetic regulation, and mitochondrial dynamics across a variety of animals under combined stress conditions. Integrating these pathways with antioxidant defenses and HSR will provide a more holistic approach to the biochemical and physiological mechanisms underlying stress adaptation. Despite these mechanistic advances, substantial methodological challenges remain for how these integrated responses can be disentangled and validated in ecologically realistic multistressor conditions. While controlled laboratory experiments allow partial isolation and manipulation of individual stressors, they often oversimplify the complexity and variability that characterize natural ecosystems. In contrast, field experiments provide greater ecological realism but inherently limit the ability to unravel the effects of individual environmental drivers, as stressors such as temperature, oxygen availability, salinity, pH, food availability, and pollution typically co-occur and interact simultaneously. Approaches such as translocation or reciprocal transplant experiments [62] can partially overcome this limitation by exposing organisms to contrasting environmental conditions while controlling population background. Fully isolating specific abiotic stressors in the field remains challenging, although some perturbation is possible, such as shading or manipulating substrate color to perturb temperature [318], or filters to control UV radiation [17,69]. Future progress will depend on integrative experimental frameworks that combine the mechanistic resolution of laboratory studies with the ecological relevance of field observations, enabling a more robust understanding of organismal responses to complex and constantly changing environmental conditions.

**Figure 5:**
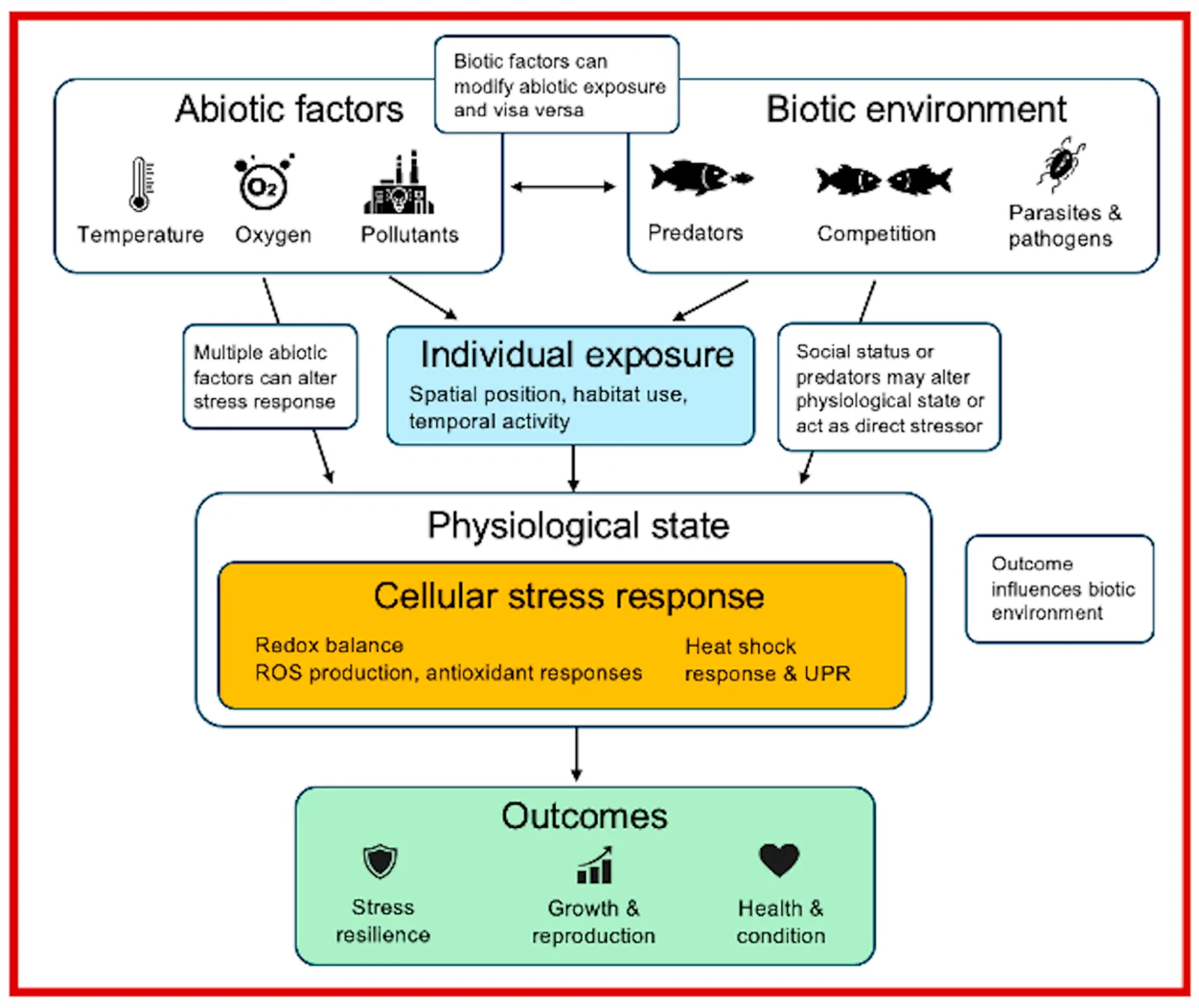
Schematic representation of integration of abiotic and biotic stressors.

A further layer of complexity in field settings is the biotic environment which interacts with abiotic stress in a way that is rarely accounted for in multistress studies. As discussed in Section 7, the social environment warrants greater integration into multistressor frameworks, both as direct stressors or modulators of an individual’s physiological response to abiotic stress (Figure 5). Further, density- and competition-dependent mechanisms can influence an individual’s spatial position, which in turn can affect abiotic stress exposure. This interplay between biotic and abiotic stress remains underexplored and represents promising avenues for future research. Linking molecular and physiological mechanisms with ecological and social context will move us closer to understanding “How Animals Work”—quoting here the classic book by Schmidt-Nielsen [319]—in terms of stress adaptation to environmental challenges.

## Figures and Tables

**Table 4:** Hsp family member expression in different species under the effect of natural environmental conditions.

Species	Stressor	Stressor Effect	Hsp Member	Reference(s)
*M. modiolus*	Temperature	Increase in winter	Hsp70	[185]
*M. galloprovincialis*	Temperature	Increase in summer	Hsp70 Hsp90	[186]
*M. trossulus*	Temperature Intertidal site	Increase in summer	Hsp70	[187]
*H. lucorum*	Temperature	Increase in summer	Hsp70 Hsp90	[188]
*P. elegans*	Temperature	Seasonally unchanged	Hsp70	[189]
*C. abyssorum tatricus*	UV-R	Increase in winter	hsp70 gene	[190]
*M. cephalus*	Temperature Pollution	Increase in summer and polluted site	mtHsp70	[191]
*M. cephalus*	Temperature Pollution	Increase in summer	Hsp90α	[192]
*S. aurata*	Temperature	Increase in spring	Hsp70 Hsp90	[54]
*P. pagrus*	Temperature	Increase in spring	Hsp70 Hsp90	[193]
*P. ridibundus*	Temperature Hibernation	Increase in spring	Hsp70 Hsp90	[194]

## Data Availability

No new data were created or analyzed in this study. Data sharing is not applicable to this article.
